# Immunoresponsive Gene 1–Itaconate Exacerbates Hypertension by Inhibiting the Cystathionine Gamma-Lyase/Hydrogen Sulfide Pathway

**DOI:** 10.3390/jcdd13070338

**Published:** 2026-07-20

**Authors:** Minglu Ma, Chenyu Fan, Shuping Han, Hu Wang, Yuzhou Xue, Boyang Lv, He Huang, Hongfang Jin, Ling Jin, Jian Liu

**Affiliations:** 1 Department of Cardiology, Peking University People’s Hospital, Beijing 100044, China; 2211110387@alumni.pku.edu.cn; 2 Department of Cardiology and Institute of Vascular Medicine, Peking University Third Hospital, Beijing 100191, China; meetfcy@stu.pku.edu.cn (C.F.);; 3 State Key Laboratory of Vascular Homeostasis and Remodeling, NHC Key Laboratory of Cardiovascular Molecular Biology and Regulatory Peptides, Beijing Key Laboratory of Cardiovascular Receptors Research, Peking University, Beijing 100871, China; 4 Laboratory of Integrative Physiology, Institute of Genetics and Developmental Biology, Chinese Academy of Sciences, Beijing 100101, China; 5 Department of Pediatrics, Peking University First Hospital, Beijing 100034, China; 6 Engineering Research Center of Techniques and Instruments for Diagnosis and Treatment of Congenital Heart Disease, Ministry of Education, Institute for Cardiovascular Development and Regenerative Medicine, Xinhua Hospital Affiliated to Shanghai Jiao Tong University School of Medicine, Shanghai 200092, China

**Keywords:** immunoresponsive gene 1, itaconate, hypertension, vascular remodeling, cystathionine gamma-lyase, hydrogen sulfide

## Abstract

The role of immunoresponsive gene 1 (IRG1)–itaconate (ITA) in hypertension remains poorly understood. This study aimed to investigate this role and the underlying mechanisms. IRG1 expression and ITA levels were assessed by Western blotting, targeted metabolomics, and ELISA. We employed global *Irg1* (mouse gene coding IRG1)-knockout mice, smooth muscle cell (SMC)-specific *Irg1* overexpression mice, and mice receiving intraperitoneal ITA injection. Hypertension was induced by angiotensin II (Ang II) infusion. Blood pressure was measured by tail-cuff method and radiotelemetry, while vascular structure and function were evaluated by histology, vascular ring assays, and Western blotting. The target proteins of ITA were identified through thermal proteomic profiling, cellular thermal shift assay, surface plasmon resonance, and molecular docking. IRG1 expression and ITA levels were significantly upregulated in mouse aortas and vascular smooth muscle cells (VSMCs) under hypertensive conditions. *Irg1* knockout markedly attenuated Ang II-induced hypertension and vascular remodeling, whereas SMC-specific *Irg1* overexpression or exogenous ITA exacerbated these pathological phenotypes. Mechanistically, ITA directly bound to cystathionine gamma-lyase (CTH) at cysteine 229, inhibiting its enzymatic activity and hydrogen sulfide (H_2_S) production. These findings demonstrate that IRG1–ITA promotes hypertension and vascular remodeling by directly targeting CTH and suppressing H_2_S production, suggesting a novel therapeutic target.

## 1. Introduction

Hypertension is a common chronic disorder characterized by elevated systemic arterial blood pressure [1]. As a major risk factor for cardiovascular disease (CVD) and cerebrovascular disease (CBD), it induces functional and structural alterations in the heart, brain, kidneys, and vasculature, ultimately leading to stroke, myocardial infarction, heart failure, and chronic kidney disease [1,2]. Owing to low awareness, low treatment rates, and the presence of resistant hypertension, many patients fail to achieve adequate blood pressure control [2]. Thus, exploring novel mechanisms and identifying effective therapeutic targets remain critically important. Vascular remodeling, a hallmark of hypertension, is characterized by medial thickening, excessive collagen deposition, and phenotypic switching of vascular smooth muscle cells (VSMCs) [3,4]. Under hypertensive conditions, metabolic reprogramming occurs in multiple cell types within the vessel wall [5,6]. Of note, metabolic reprogramming directly drives angiotensin II (Ang II)-induced vascular remodeling, and targeted interventions against this process have been shown to effectively mitigate pathological changes [7]. Therefore, hypertension-associated metabolic alterations warrant further investigation [8,9].

On the other hand, the tricarboxylic acid (TCA) cycle serves as the final common pathway and metabolic hub for carbohydrates, lipids, and amino acids, playing a vital role in maintaining systemic metabolic homeostasis [10]. Recent studies have established a clear link between TCA cycle imbalance and hypertension. For instance, both fumarate hydratase (FH) and its substrate fumarate participate in the pathogenesis of salt-sensitive (SS) hypertension [11]. Additional TCA cycle metabolites have also been implicated. Succinate promotes hypertension via succinate receptor 1 (SUCNR1)-mediated renin release [12,13]. Malate and aspartate increase L-arginine and nitric oxide production, thereby reducing blood pressure [14]. Alpha-ketoglutarate is positively associated with salt-sensitive hypertension in Chinese populations [15]. Moreover, lower urinary citrate excretion is independently associated with hypertension prevalence [16]. Despite these findings, the potential role of other TCA cycle-derived metabolites—such as itaconate (ITA)—in blood pressure regulation remains largely unexplored.

ITA has recently gained attention for its role in various diseases [17]. In activated macrophages, ITA is produced via decarboxylation of cis-aconitate, a reaction catalyzed by the rate-limiting enzyme immunoresponsive gene 1 (IRG1, also known as ACOD1) [18]. Early studies on the IRG1–ITA axis focused primarily on immune cells and diseases such as infection, immune disorders, and cancer, highlighting its antibacterial, anti-inflammatory, and antioxidant functions [19,20]. In CVD, IRG1-derived ITA exerts protective immunomodulatory effects in ischemic heart disease, valvular heart disease, and abdominal aortic aneurysm (AAA) [21,22]. However, its role in atherosclerosis remains controversial, with both protective and detrimental effects reported [23,24]. Notably, the latter study suggests that the IRG1–ITA axis may influence disease progression through previously unrecognized non-immune mechanisms [24]. Nevertheless, whether the IRG1–ITA axis contributes to hypertension has not yet been explored. Based on these findings, we hypothesized that the IRG1–ITA axis is involved in the pathogenesis of hypertension, although its beneficial or detrimental nature remains to be determined.

To investigate potential upstream regulators of blood pressure control, we turned to hydrogen sulfide (H_2_S), an endogenous gasotransmitter critical for vascular homeostasis [25]. In mammals, H_2_S is endogenously generated by three principal enzymes: cystathionine gamma-lyase (CTH, also known as CSE), cystathionine beta-synthase (CBS), and 3-mercaptopyruvate sulfurtransferase (3-MST) [26]. Among them, CTH is considered the predominant H_2_S-producing enzyme in the aorta, whereas CBS and 3-MST contribute predominantly to H_2_S production in extravascular tissues such as the brain, liver, kidney, and nervous system, with 3-MST being localized to mitochondria [27,28]. Plasma H_2_S levels are reduced in hypertensive patients and animal models; *Cth*-deficient mice develop age-dependent hypertension; and exogenous H_2_S supplementation lowers blood pressure [29,30,31]. Mechanistically, H_2_S induces vasodilation via multiple pathways, including K_ATP_ channel activation, interaction with the NO-cGMP pathway, inhibition of mitochondrial complexes I and III, and its function as an adipocyte-derived relaxing factor [32,33,34]. Additionally, H_2_S suppresses VSMC proliferation, as evidenced by increased proliferation in *Cth*-deficient mice and dose-dependent inhibition by the H_2_S donor NaHS [35]. Although the downstream mechanisms of the CTH/H_2_S pathway are relatively well understood, its upstream regulation—particularly by small-molecule metabolites—remains largely unknown [36,37,38,39]. We therefore hypothesized that the IRG1–ITA axis may regulate blood pressure and vascular remodeling, possibly through modulation of the CTH/H_2_S pathway.

In this study, we investigated the role of the IRG1–ITA axis in hypertension using an Ang II-induced hypertensive mouse model and Ang II-stimulated human aortic smooth muscle cells (HASMCs), focusing on blood pressure elevation, vascular remodeling, and VSMC phenotypic switching. We found that the IRG1–ITA axis drives hypertensive vascular pathology by inhibiting the CTH/H_2_S pathway, thereby exacerbating hypertension. These results identify a previously unrecognized mechanism and suggest that targeting IRG1 may represent a novel therapeutic strategy for hypertension.

## 2. Materials and Methods

### 2.1. Animal Procedures

Male, 8–10 weeks, 20–25 g, C57BL/6J wild-type (WT) mice were purchased from the Department of Laboratory Animal Science, Peking University Health Science Center. *Irg1* knockout (*Irg1^−/−^*) mice and WT (*Irg1^+/^^+^*) littermate mice on a C57BL/6 background were obtained from Cyagen Biosciences Inc. (Suzhou, China; stock #S-KO-02680). To generate smooth muscle cell (SMC)-specific *Irg1* overexpression mice, an adeno-associated virus serotype 9 (AAV9) vector carrying the mouse α-Smmhc promoter was constructed and packaged. AAV9 encoding mouse *Irg1* was produced by Hanheng Biotechnology (Shanghai, China). Mice received a tail vein injection of 1.5 × 10^11^ vector genomes (vg) of either AAV9-α-Smmhc-null or AAV9-α-Smmhc-*Irg1*. Two weeks after viral injection, mice were anesthetized with 2% isoflurane for subsequent experiments. To establish a hypertensive mouse model, Ang II (1000 ng/kg/min; Sigma-Aldrich, St. Louis, MO, USA, A9525) was infused continuously for two weeks via subcutaneously implanted osmotic pumps (Alzet, Model 1002; DURECT Corporation, Cupertino, CA, USA). For ITA administration, ITA (Sigma-Aldrich, St. Louis, MO, USA, I29204) was dissolved in DMSO and further diluted with sterile saline prior to intraperitoneal injection at a dose of 75 mg/kg every two days, a dosage regimen referenced from previous animal studies [40]. All ITA injections were synchronized with the 14-day Ang II or saline infusion period, resulting in a total of 7 injections per mouse. ITA treatment was administered to two separate WT groups: one receiving saline infusion and the other receiving Ang II infusion. Corresponding vehicle control groups received an equal volume of saline at identical time points to match the injection frequency of ITA-treated groups. Mice were randomised to groups using random number tables. Procedures were randomized, and outcome assessments were blinded. All procedures were performed in the SPF animal facility at Peking University Health Science Center. In accordance with the 3Rs (Replacement, Reduction, and Refinement) principles of animal welfare, tissues harvested from individual mice were reused for multiple downstream assays wherever practicable. A total of 101 mice were used across all experiments; detailed group-wise allocation for different experimental readouts is summarized in Supplementary Table S1.

All animal experiments complied with the Guidelines for the Care and Use of Laboratory Animals and were approved by the Institutional Animal Care and Use Committee (IACUC) of Peking University Health Science Center (approval No.: BCAJ0266).

### 2.2. ITA Detection

The levels of ITA in mouse plasma and HASMCs were determined using targeted central carbon metabolomics, and the detection was performed by Bioprofile (Shanghai, China). ITA levels in the aortas from mice were measured using the ITA ELISA kit (Huabo Deyi, Beijing, China, HBDY-927922O1). This dual-platform approach was adopted because metabolomics enables absolute quantification of small metabolites in fluid and cellular samples, whereas the limited amount of aortic tissue available per mouse necessitated an ELISA platform that consumes less tissue, thereby preserving material for other biochemical assays.

For plasma metabolomics, blood was collected from the retro-orbital venous plexus using EDTA-coated tubes and centrifuged at 3000 rpm for 15 min at 4 °C. The upper plasma layer was retained, snap-frozen in liquid nitrogen, and stored at −80 °C until analysis. Approximately 100 µL of plasma per mouse was used for metabolomics. For aortic ITA measurement, 0.01–0.02 g aortic tissue from each mouse was homogenized in extraction buffer using a tissue grinder. After centrifugation, the supernatants were collected, and ITA levels were determined using a competitive antigen-HRP ELISA kit with high specificity for ITA, following the manufacturer’s protocol. For cellular ITA measurement, approximately 1 × 10^6^ HASMCs were required per sample. Cells were harvested on ice, washed with ice-cold PBS, scraped, pelleted by centrifugation, snap-frozen in liquid nitrogen, and stored at −80 °C until analysis.

### 2.3. Blood Pressure Monitoring

Murine blood pressure was measured in accordance with the recommendations of the Council on High Blood Pressure Research of the American Heart Association [41]. Non-invasive measurements were performed using the BP-2010A system (RWD Life Science Co., Ltd., Shenzhen, China) and the CODA tail-cuff system (Kent Scientific, Torrington, CT, USA). Baseline blood pressure and blood pressure at 3, 7, 10, and 14 days after Ang II infusion were recorded. Five readings were averaged for each mouse at each time point.

Meanwhile, radiotelemetry probes (Model PA-C10, Data Sciences International, St. Paul, MN, USA) were implanted into the left carotid artery of each mouse under anesthesia. After a 10-day recovery period, osmotic minipumps containing Ang II were implanted. 24 h continuous blood pressure monitoring was conducted 13 days after minipump implantation using the Dataquest ART Silver acquisition system (Data Sciences International). Raw data were recorded every 10 s, and mean blood pressure values were calculated every hour for the construction of 24 h blood pressure profiles.

### 2.4. Histological Analysis

Aortas were harvested, fixed in 4% paraformaldehyde, dehydrated, and then embedded in paraffin. Tissues were cut into 5-μm-thick sections. Sections were incubated at 68 °C for 1 h, deparaffinized in xylene, and then stained with hematoxylin-eosin (HE) (Solarbio, Beijing, China, G1120) and Masson trichrome (Solarbio, G1340). Aortic media thickness and collagen deposition percentage were quantified using Image-Pro Plus software (Version 6.0, Media Cybernetics, Inc., Rockville, MD, USA).

### 2.5. Assessment of Mesenteric Arterial Tone

Isometric tension measurements were performed using a wire myograph system (Model 620M, Danish Myo Technology A/S, Aarhus Nord, Denmark) [42]. Mouse mesenteric arteries were rapidly isolated, cleaned of adipose tissue, and cut into approximately 1 mm segments. Each segment ring was mounted in an organ chamber containing Krebs solution (119 mmol/L NaCl, 25 mmol/L NaHCO_3_, 4.7 mmol/L KCl, 1.2 mmol/L KH_2_PO_4_, 2.5 mmol/L CaCl_2_, 1 mmol/L MgCl_2_, and 11 mmol/L D-glucose; pH 7.35–7.45) bubbled with 95% O_2_ and 5% CO_2_, and equilibrated for 1 h. Stock solutions of phenylephrine (Phe; Solarbio, Cat. No. IP3150), acetylcholine (ACh; Solarbio, Cat. No. A8910), Nω-Nitro-L-arginine methyl ester hydrochloride (L-NAME; Solarbio, Cat. No. N8630) and sodium nitroprusside (SNP; Solarbio, Cat. No. S9560) were separately prepared using sterile Milli-Q ultrapure water. Viability was confirmed with 60 mmol/L KCl. For vasoconstriction detection, cumulative Phe was administered at concentrations of 1 nmol/L, 3 nmol/L, 10 nmol/L, 30 nmol/L, 100 nmol/L, 300 nmol/L, 1 μmol/L, 3 μmol/L, and 10 μmol/L. For endothelium-dependent vasodilation detection, arterial rings were pre-contracted with 3 μmol/L Phe. Cumulative ACh was then added at concentrations of 1 nmol/L, 3 nmol/L, 10 nmol/L, 30 nmol/L, 100 nmol/L, 300 nmol/L, 1 µmol/L, 3 µmol/L, and 10 µmol/L. For endothelium-independent vasodilation detection, arterial rings were pre-incubated with 100 µmol/L L-NAME for 30 min, followed by pre-contraction with 3 µmol/L Phe. Cumulative SNP was applied at concentrations of 1 nmol/L, 3 nmol/L, 10 nmol/L, 30 nmol/L, 100 nmol/L, 300 nmol/L, 1 µmol/L, 3 µmol/L, and 10 µmol/L. After each cumulative dose–response test, the organ bath buffer was replaced, and arterial rings were washed four times with Krebs solution. Subsequent drug treatments were performed only after vascular tension returned to a stable baseline. Vascular contractile and relaxant responses were expressed as changes in force normalized to the length of each arterial segment. Normalized active force was calculated as ΔF_norm = (F_active − F_relax)/L, where F_active is the force recorded during pharmacological stimulation, F_relax is the baseline resting force, and L is the vessel segment length (mm). The same normalization method was applied consistently to all vessels and experimental groups. Vasorelaxation magnitude was defined as the absolute reduction in normalized active force, and overall responses were quantified by calculating the area under the log concentration-response curve (AUC) for each artery. EC_50_ values were not calculated, as the study aimed to evaluate overall vasodilatory capacity rather than drug potency.

### 2.6. Immunofluorescence Staining

Mouse aortas were fixed in 4% paraformaldehyde, cryoprotected in 20% sucrose, and then embedded in Tissue Tek^®^ O.C.T. compound (Sakura Finetek, Torrance, CA, USA). Frozen sections (10-μm-thick) were permeabilized with 0.1% Triton X-100 for 10 min, followed by blocking with 5% bovine serum albumin (BSA) at room temperature for 20 min. Subsequently, the sections were incubated overnight (16 h) at 4 °C with the following primary antibodies: mouse anti-α-SMA antibody (Abcam, Cambridge, UK, ab7817) and rabbit anti-IRG1 antibody (CST, Danvers, MA, USA, 17805). Secondary antibodies (Alexa Fluor 488-conjugated anti-rabbit, Abcam, ab150077; Alexa Fluor 568-conjugated anti-mouse, Abcam, ab175473) were applied at a 1:400 dilution for 60 min at room temperature. Nuclei were counterstained with DAPI (Sigma, F6057). Images were captured using a confocal microscope (Model LSM 800, Carl Zeiss, Oberkochen, Germany) and analyzed with analyzed with ZEN (blue edition) software (version 3.4; Carl Zeiss, Oberkochen, Germany).

### 2.7. Western Blot Analysis

Total proteins were extracted from aortic tissue and cells using radioimmunoprecipitation assay (RIPA) buffer supplemented with a protease/phosphatase inhibitor cocktail (CST, 5872). Total protein concentration was quantified with a Pierce BCA Protein Assay Kit (Thermo Fisher Scientific, Waltham, MA, USA, 23225). Protein samples (15 µg per lane) were separated by SDS-PAGE (Biotides, Beijing, China, WB1102) and transferred at a constant current of 400 mA for 45 min to nitrocellulose membranes (Cytiva, Marlborough, MA, USA, 10600001). Membranes were blocked with 5% non-fat dry milk in TBST for 1 h and incubated with primary antibodies diluted in Western Primary Antibody Dilution Buffer (Beyotime, Shanghai, China, P0023A) overnight (12–16 h) at 4 °C. After three 5 min washes with TBST, membranes were incubated with HRP-conjugated secondary antibodies diluted in TBST for 1 h at room temperature. Protein bands were visualized using enhanced chemiluminescence (ECL) reagent (Millipore, Billerica, MA, USA, WBKLS0500) and GeneSys software (v1.6.3.0).

The primary antibodies used in this study included: anti-mouse IRG1 (CST, 17805, 1:1000), anti-human IRG1 (CST, 77510, 1:1000), anti-CTH (Abcam, ab151769, 1:2000), anti-CBS (Abcam, ab313382, 1:1000), anti-COL1A1 (Abcam, ab260043, 1:3000), anti-α-SMA (Abcam, ab7817, 1:3000), anti-SM22α (Abcam, ab14106, 1:3000), anti-GAPDH (CST, 5174, 1:5000), and anti-β-actin (Proteintech, Rosemont, IL, USA, 66009-1-Ig, 1:5000). HRP-conjugated goat anti-mouse IgG (Proteintech, SA00001-1, 1:5000) and HRP-conjugated goat anti-rabbit IgG (Proteintech, SA00001-2, 1:5000) were used as secondary antibodies, except for GAPDH and β-actin, which were detected at a secondary antibody dilution of 1:10,000. Densitometry was performed using ImageJ software (version 1.53k; National Institutes of Health, Bethesda, MD, USA).

### 2.8. Cell Culture and Treatment

HASMCs (BNCC354548, BeNa Culture Collection, Beijing, China) were maintained in HASMC culture medium (BNCC354731, BeNa Culture Collection, Beijing, China) containing 50 U/mL penicillin plus 50 μg/mL streptomycin and 10% fetal bovine serum (FBS) at 37 °C with 5% CO_2_. Cells were treated with Ang II (1 × 10^−6^ M; Sigma, A9525) with or without ITA (1 mM; Sigma, I29204) for 48 h. For knockdown experiments, HASMCs were infected with recombinant adenoviruses expressing shRNA targeting IRG1 (sh-*IRG1*) or a negative control (sh-Control), constructed by Hanheng Biotechnology (Shanghai, China). The sh-IRG1 sequence was as follows:

Sense:5′-TCGAGGCAGTTCCAACATATCCAGCACTGTTCTCGAGAACAGTGCTGGATATGTTGGAACTGTTTTTTA-3′;

Antisense:5′-AGCTTAAAAAACAGTTCCAACATATCCAGCACTGTTCTCGAGAACAGTGCTGGATATGTTGGAACTGCC-3′.

### 2.9. Cell Counting Kit-8 (CCK-8) Assay

HASMCs were seeded into 96-well plates at a density of 5 × 10^3^ cells per well and starved in serum-free medium for 12 h. Then, the culture medium was replaced with the conditioned medium. After 24 h of treatment, cell proliferation was evaluated using a CCK-8 kit (Dojindo Laboratories, Kumamoto, Japan, CK04). CCK-8 solution was added to each well at a ratio of 1:10, followed by incubation at 37 °C in the dark for 2 h. The absorbance was measured at a wavelength of 450 nm.

### 2.10. Wound Healing Assay

Cell migration ability was examined using the wound healing assay in 96-well plates (Corning, NY, USA, 3599). HASMCs were seeded at 3 × 10^4^ cells per well. When cells reached 95% confluence in the wells, a standardized scratching tool was used to create a scratch in each well. After washing with PBS, the medium was replaced with conditioned medium devoid of serum and growth factors. Automated image acquisition was performed using the Incucyte^®^ Live-Cell Analysis System (Sartorius, Ann Arbor, MI, USA), and the extent of wound healing was quantitatively analyzed at 0 h and 12 h.

### 2.11. Thermal Proteome Profiling (TPP)

HASMCs were lysed via eight freeze–thaw cycles and centrifuged (20,000× *g*, 1 h, 4 °C). The protein concentration of the supernatant was measured using a BCA protein assay kit. Afterwards, cell lysates at 2 mg/mL were incubated with 1 mM ITA or dimethyl sulfoxide (DMSO, vehicle control) at 25 °C for 1 h, aliquoted, and heated at 37 °C or 73 °C for 4 min. After centrifugation (20,000× *g*, 30 min, 4 °C), the supernatant was subjected to LC-MS/MS analysis [43]. This experiment was performed by Nanjing Chomix Biotechnology Co., Ltd. (Nanjing, China).

### 2.12. CETSA

HASMCs and HEK293A cells transfected with CTH-WT or mutant constructs were harvested at 80% confluence. Total cellular proteins were extracted using NP-40 cell lysis buffer supplemented with a protease and phosphatase inhibitor cocktail. Protein concentrations of all lysates were quantified via BCA assay and normalized to a uniform concentration of 2 mg/mL. Normalized lysates were incubated with 1 mM ITA or DMSO under gentle rotation at room temperature (RT) for 1 h, then heated at specific temperatures for 4 min. For HASMC CETSA, samples were heated at 37, 52, 57, 62, 67, 72, and 77 °C; for HEK293A cells, samples were heated at 37, 62, 67, 72, and 77 °C. After centrifugation, the supernatants were collected and subjected to Western blot analysis with 15 µg of protein per lane to detect CTH protein levels.

### 2.13. SPR

Binding kinetics between ITA and its potential protein target CTH were analyzed using a Biacore S200 system (Cytiva, Marlborough, MA, USA). Purified recombinant CTH protein (MCE, Monmouth Junction, NJ, USA, HY-P70025) with a stock concentration of 1.42 mg/mL was diluted 1:20 with running buffer and immobilized onto a CM5 Series S sensor chip (Cytiva, BR-1005-30). ITA was serially diluted (0.00019–0.2 mM) and flowed over a CM5 Series S sensor chip at a flow rate of 30 μL/min (association: 120 s, dissociation: 240 s) at 25 °C. Experimental data were processed and analyzed using Biacore^TM^ Insight Software (version 4.0.8.19879; Cytiva), with reference to a blank flow cell. Kinetics were fitted to a 1:1 Langmuir binding model to determine the association rate constant (k_a_), dissociation rate constant (k_d_) and equilibrium dissociation constant (K_D_).

### 2.14. Molecular Docking

Molecular docking was performed using AutoDock Vina (version 1.1.2; The Scripps Research Institute, La Jolla, CA, USA). The three-dimensional structure of human CTH was retrieved from the Protein Data Bank (PDB) and prepared by removing water molecules and adding hydrogen atoms. The ITA molecule was geometrically optimized at the DFT/B3LYP/6-311G* level. The binding grid box was centered on the predicted active pocket of CTH.

### 2.15. Plasmid Transfection

HEK293A cells were cultured in Dulbecco’s modified Eagle’s medium (DMEM) supplemented with 10% FBS and 50 U/mL penicillin and 50 μg/mL streptomycin. WT or mutant (Cys229Mut, Asn228Mut, and Ser231Mut) human CTH coding sequences were cloned into the pcDNA3.1 expression vector (Genomeditech, Shanghai, China). Plasmid transfection was performed using Lipofectamine 3000 reagent (Invitrogen, Carlsbad, CA, USA, L3000015) according to the manufacturer’s protocol (1 µg of plasmid DNA per well in a 6-well plate).

### 2.16. Detection of H_2_S Level and CTH Enzyme Activity

CTH enzymatic activity and H_2_S concentrations in mouse aortic tissue and HASMCs were separately quantified according to the corresponding kit manuals. CTH activity was detected with a CTH Activity Assay Kit (Zike Bio, Shenzhen, China, Cat. No. GMS50550.1). Briefly, ~0.1 g fresh mouse aortic tissue was homogenized in ice-cold lysis buffer, and 1 × 10^7^ logarithmic-phase HASMCs per replicate were harvested, pelleted and resuspended in lysis buffer; both tissues and cells were incubated on ice for 30 min for full lysis, then centrifuged at 13,000 × *g* for 10 min at 4 °C to collect clear supernatants. For aortic tissue lysates, the concentration was adjusted to 5 mg/mL; for HASMC lysates, to 2 mg/mL. Equal volumes of the adjusted lysates (20 µL per well) were loaded into 96-well plates for background control and activity detection. After 30 min enzymatic reaction at 37 °C and 5 min chromogenic incubation, absorbance was recorded at 412 nm to calculate net CTH activity, which was normalized to total protein and presented as U/mg protein. For H_2_S measurement, tissue and cell supernatants were prepared as described above and detected using an H_2_S Content Detection Kit (Solarbio, Cat. No. BC2055). After a 20 min light-shielded reaction at room temperature, absorbance was read at 680 nm. H_2_S levels were calculated based on standard curves, with aortic tissue results normalized to tissue weight and cell sample results normalized to total protein content.

### 2.17. Plasma Cysteine Measurement

Plasma cysteine (Cys) concentrations in mice were determined using a commercial assay kit (Solarbio, Cat. No. BC0185) according to the manufacturer’s microplate protocol. Plasma collection was performed as described above. For plasma pretreatment, 0.1 mL of mouse plasma was thoroughly mixed with 0.15 mL of extraction buffer (provided in the kit) and centrifuged at 11,000 rpm for 10 min at 4 *°C*. Sample absorbance was verified to fall within the linear range of the standard curve (0.03125*–*3 μmol/mL); samples exceeding this range were appropriately diluted with extraction buffer, and the dilution factor was recorded for final concentration calculation; the supernatant was collected on ice for subsequent analysis. Aliquots of 40 μL of processed plasma supernatant, standard working solutions, or distilled water (blank) were dispensed into corresponding wells of a 96-well plate, followed by addition of the detection reagents. After gentle mixing, the plate was incubated at room temperature for 15 min, and absorbance was recorded at 600 nm. The Cys concentration in the extracted supernatant was calculated from the standard curve.

### 2.18. Immunohistochemistry for CTH in Aortic Sections

Paraffin-embedded mouse aortic tissues were sectioned at 5 µm. Sections were baked at 68 °C for 1 h, deparaffinized in xylene, and rehydrated through a graded ethanol series to distilled water. Antigen retrieval was performed in citrate antigen retrieval buffer (pH 6.0) by heating at 95 °C for 15 min. Endogenous peroxidase activity was quenched with 3% hydrogen peroxide for 15 min at room temperature. After nonspecific binding was blocked with 5% normal goat serum for 30 min at room temperature, sections were incubated overnight at 4 °C with anti-CTH antibody (Abcam, ab151769; 1:500 dilution). After washing with PBS, sections were incubated with HRP-conjugated goat anti-rabbit IgG (Proteintech, SA00001-2; 1:500 dilution) for 1 h at room temperature. Immunoreactivity was visualized with 3,3′-diaminobenzidine (DAB), followed by hematoxylin counterstaining. Sections were then dehydrated, cleared, and mounted. Images were acquired under identical settings across comparable groups. CTH staining in the aortic media was quantified as mean optical density using Image-Pro Plus software (Version 6.0) by an investigator blinded to group allocation.

### 2.19. Statistical Analysis

Statistical analyses were performed using GraphPad Prism 9 (version 9.5.1; GraphPad Software, San Diego, CA, USA). Data normality was assessed using the Shapiro–Wilk test. Normally distributed data are presented as mean ± standard error of the mean (SEM) and were analyzed using Student’s *t*-test, one-way ANOVA, or two-way ANOVA followed by Tukey’s post hoc test as appropriate. For CETSA data, two-way ANOVA followed by Bonferroni’s post hoc test was applied. Data that did not pass the normality test are presented as median (interquartile range) and were analyzed using the Mann–Whitney U test for two-group comparisons or the Kruskal–Wallis test followed by Dunn’s multiple-comparisons test for comparisons among three or more groups. We considered *p* < 0.05 to be statistically significant.

## 3. Results

### 3.1. ITA Is Upregulated in Aortas from Hypertensive Mice and Ang II-Stimulated HASMCs

**Figure 1:**
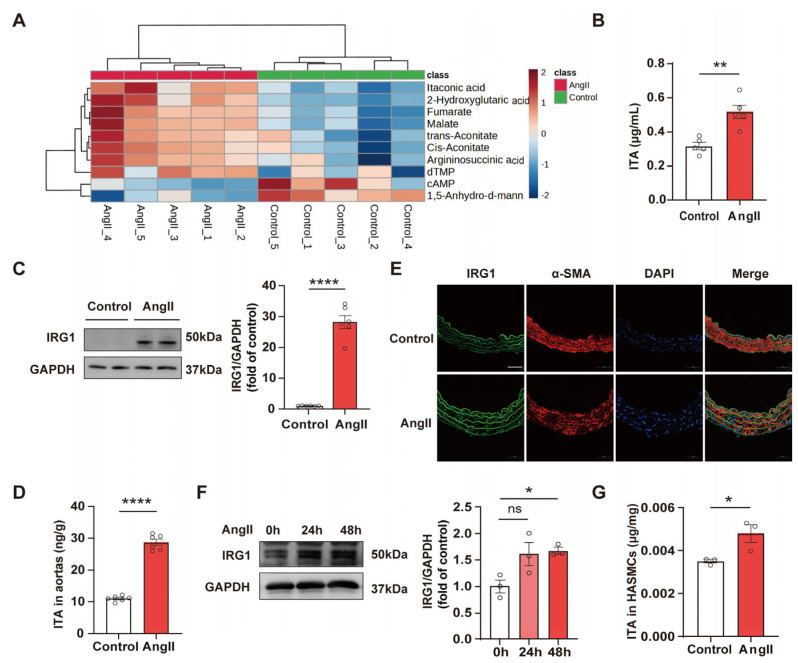
IRG1 and ITA are upregulated in aortic VSMCs during hypertension. (**A**) Central carbon metabolomic profiling of plasma from hypertensive and normotensive mice (*n* = 5 per group). A heatmap was generated using MetaboAnalyst 6.0. (**B**) Absolute levels of ITA in plasma from hypertensive and normotensive mice (*n* = 5 per group). (**C**) IRG1 protein expression in the aortas of normotensive and hypertensive mice (*n* = 6 per group). (**D**) Aortic ITA levels in normotensive and hypertensive mice measured by ELISA (*n* = 6 per group). (**E**) Representative immunofluorescence images of IRG1 (green) and α-SMA (red) in mouse aortic tissues. Nuclear staining was performed with DAPI (blue). Scale bar = 50 μm. (**F**) Representative Western blots and statistical analysis of IRG1 expression in HASMCs treated with Ang II (1 × 10^−6^ M) for 0 h, 24 h, and 48 h (*n* = 3 per group). (**G**) ITA levels in HASMCs before and after Ang II stimulation (48 h) were determined by targeted central carbon metabolomics (*n* = 3 per group). Data are presented as the mean ± standard error of the mean (SEM). Two-group comparisons were performed using Student’s *t*-test, and multiple-group comparisons were performed using one-way ANOVA. ns, *p* > 0.05; * *p* < 0.05; ** *p* < 0.01; **** *p* < 0.0001. Abbreviations: ITA: itaconate; IRG1: immunoresponsive gene 1; GAPDH: Glyceraldehyde-3-Phosphate Dehydrogenase; α-SMA: α-smooth muscle actin; VSMC: vascular smooth muscle cell; HASMC: human aortic smooth muscle cell.

Central carbon metabolomic analysis of plasma from Ang II-induced hypertensive mice revealed that plasma ITA was significantly upregulated in hypertensive mice compared with controls (*p* < 0.01, Figure 1A,B). Given that vascular remodeling occurs locally in the vessel wall, we hypothesized that the systemic elevation of ITA levels might be partially attributable to its local upregulation in the aorta. Since IRG1 is the only rate-limiting enzyme catalyzing ITA synthesis, we examined IRG1 protein expression by Western blotting and measured ITA levels in mouse aortas by enzyme-linked immunosorbent assay (ELISA). Both IRG1 protein expression and ITA levels were significantly upregulated in the aortas from hypertensive mice (*p* < 0.0001, Figure 1C,D), suggesting localized activation of the key enzyme IRG1 within the vessel wall and supporting a local vascular contribution to ITA production.

Given that VSMCs are primary drivers of vascular remodeling and constitute the major structural cell type in the aorta, we focused on this population to investigate the cellular source responsible for the upregulation of IRG1 and ITA within the vessel wall tissue under hypertensive conditions. We performed immunofluorescence staining on frozen sections of mouse thoracic aorta tissue and found that IRG1 expression was upregulated in aortic smooth muscle cells (SMCs) of hypertensive mice (Figure 1E). We next validated the above findings in vitro. Ang II stimulation led to a significant upregulation of IRG1 protein expression and elevated ITA levels in HASMCs (*p* < 0.05, Figure 1F,G), whereas no significant changes were observed in human umbilical vein endothelial cells (HUVECs) (Supplementary Figure S1A).

Additionally, we analyzed public transcriptomic datasets (GEO database), which indirectly validated our experimental findings. Analysis of GSE302827 showed that *Irg1* was undetectable in aortic endothelial cells (ECs) from both SS hypertensive rats and normotensive control rats (Supplementary Figure S1B). Similarly, dataset GSE211978 showed the absence of *IRG1* expression in HUVECs, regardless of Ang II stimulation (Supplementary Figure S1C). In addition, dataset GSE291516 showed that *Irg1* expression in VSMCs could be upregulated after Ang II stimulation (Supplementary Figure S1D).

These results indicate that VSMCs are a major cellular source of ITA upregulation in the hypertensive aorta, as evidenced by the activation of the key enzyme IRG1 in VSMCs during hypertension. Whether other cell types or tissues also contribute to ITA production—locally or systemically—under hypertensive conditions remains an open question for future investigation.

### 3.2. Irg1 Knockout Alleviates Ang II-Induced Hypertension and Vascular Remodeling

To investigate the role of the IRG1–ITA axis in hypertension, we used global *Irg1^−/−^* mice. Compared with *Irg1^+/^^+^* mice, IRG1 protein was undetectable in the aortas of *Irg1^−/−^* mice after Ang II stimulation (*p* < 0.0001, Supplementary Figure S2A), consistent with a complete loss of Ang II-induced ITA elevation (*p* < 0.0001, Supplementary Figure S2B). The above results collectively confirm the successful generation of *Irg1^−/−^* mice.

**Figure 2:**
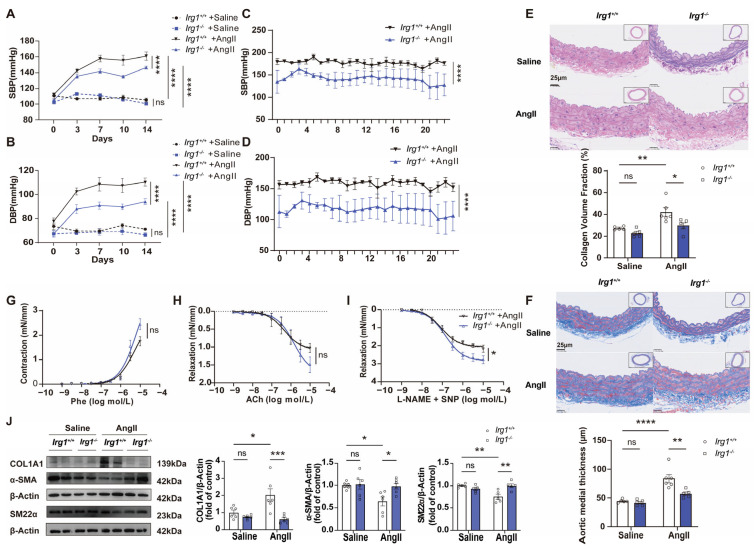
*Irg1* knockout attenuates Ang II-induced blood pressure elevation, endothelium-independent relaxation dysfunction, and vascular remodeling. (**A**,**B**) SBP (**A**) and DBP (**B**) in *Irg1^+/+^* and *Irg1^−/−^* mice infused with saline or Ang II for 14 days (*n* = 6 per group). (**C**,**D**) 24 h recordings of SBP (**C**) and DBP (**D**) in *Irg1^+/+^* and *Irg1^−/−^* mice measured by radiotelemetry after 13 days of Ang II infusion (*n* = 3 per group). (**E**,**F**) Representative H&E (**E**) and Masson’s trichrome staining (**F**) images, and statistical analysis of aortic medial thickness and collagen deposition in thoracic aortas from *Irg1^+/+^* and *Irg1^−/−^* mice treated with saline or Ang II for 14 days (*n* = 5–6 per group). Scale bar = 25 μm. (**G**–**I**) Concentration-response curves for vasoconstriction (induced by Phe) (**G**), endothelium-dependent relaxation (induced by ACh) (**H**), and endothelium-independent relaxation (induced by SNP) (**I**) in mesenteric resistance arteries from *Irg1^+/+^* and *Irg1^−/−^* mice following Ang II infusion for 14 days (*n* = 6 per group). (**J**) Expression levels of COL1A1, α-SMA, and SM22α in aortas from *Irg1^+/+^* and *Irg1^−/−^* mice infused with saline or Ang II for 14 days (*n* = 5–6 per group). Data are presented as mean ± SEM. Statistical comparisons were performed using Student’s *t*-test for two groups and two-way ANOVA with appropriate post hoc tests for multiple groups; AUC values for concentration-response curves (**G**–**I**) were compared by unpaired *t*-test. ns, *p* > 0.05; * *p* < 0.05; ** *p* < 0.01; *** *p* < 0.001; **** *p* < 0.0001. Abbreviations: SBP, systolic blood pressure; DBP, diastolic blood pressure; Ang II, angiotensin II; Phe, phenylephrine; ACh, acetylcholine; L-NAME, Nω-nitro-L-arginine methyl ester; SNP, sodium nitroprusside; COL1A1, collagen 1α1; α-SMA, α-smooth muscle actin; SM22α, smooth muscle 22α; AUC, area under the log concentration-response curve; SEM, standard error of the mean.

Under physiological conditions, systolic blood pressure (SBP) and diastolic blood pressure (DBP) measured by the tail-cuff method at multiple time points over 14 days did not differ significantly between *Irg1^+/^^+^* and *Irg1^−/−^* mice (Figure 2A,B). Under Ang II-induced hypertension, both tail-cuff method and 24 h radiotelemetry measurements revealed that *Irg1* knockout significantly attenuated Ang II-induced elevations in SBP and DBP compared with WT mice (*p* < 0.0001, Figure 2A–D). Furthermore, we observed that Ang II-induced vascular structure and function damage were milder in *Irg1^−/−^* mice compared with *Irg1^+/^^+^* mice. H&E staining and Masson’s trichrome staining were used to assess medial thickness and collagen deposition in the thoracic aorta, respectively. Histological analysis revealed that Ang II infusion significantly increased aortic medial thickness and collagen deposition in the aortas of *Irg1^+/^^+^* mice, whereas *Irg1* knockout attenuated these Ang II-induced vascular structural damages (*p* < 0.01, Figure 2E; *p* < 0.05, Figure 2F). Vascular ring assays demonstrated that *Irg1* knockout significantly improved Ang II-induced endothelium-independent vasodilatory dysfunction (*p* < 0.05, Figure 2G–I and Figure S3A–C). Moreover, *Irg1* knockout significantly inhibited the upregulation of collagen 1α1 (COL1A1), a key marker of extracellular matrix deposition (*p* < 0.001), and reversed the downregulation of α-smooth muscle actin (α-SMA) (*p* < 0.05) and smooth muscle 22α (SM22α) (*p* < 0.01), both contractile phenotype markers of VSMCs, in the aortas from hypertensive mice (Figure 2J), suggesting that *Irg1* knockout suppressed Ang II-induced conversion of VSMCs from a contractile phenotype to a synthetic phenotype.

These results demonstrate that *Irg1* knockout confers a protective effect against Ang II-induced hypertension and vascular remodeling.

### 3.3. SMC-Specific Irg1 Overexpression and Exogenous ITA Exacerbate Ang II-Induced Hypertension and Vascular Remodeling

**Figure 3:**
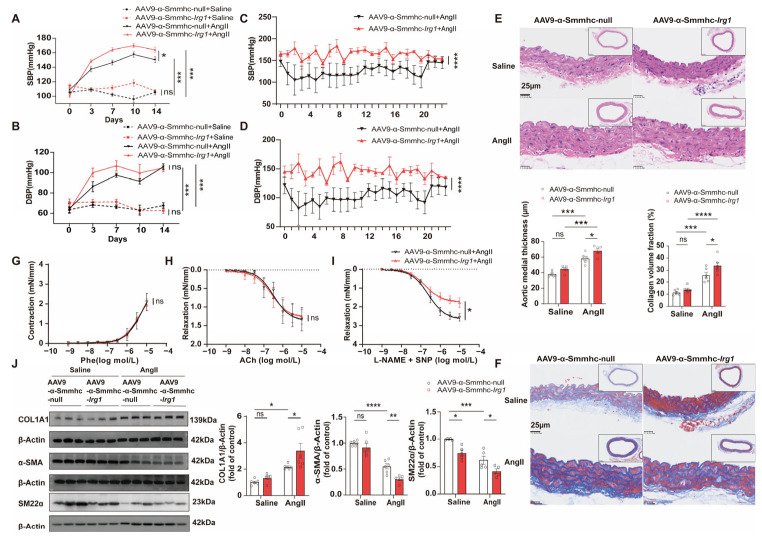
SMC-specific *Irg1* overexpression aggravates Ang II-induced blood pressure elevation, endothelium-independent relaxation dysfunction, and vascular remodeling. (**A**,**B**) SBP (**A**) and DBP (**B**) measured via the tail-cuff method in AAV9-α-Smmhc-null and AAV9-α-Smmhc-*Irg1* mice infused with saline or Ang II for 14 days (*n* = 6 per group). (**C**,**D**) 24 h recordings of SBP (**C**) and DBP (**D**) in AAV9-α-Smmhc-null and AAV9-α-Smmhc-*Irg1* mice measured by radiotelemetry after 13 days of Ang II infusion (*n* = 3 per group). (**E**,**F**) Representative H&E and Masson’s trichrome staining images, and statistical analysis of aortic medial thickness and collagen deposition in thoracic aortas from AAV9-α-Smmhc-null and AAV9-α-Smmhc-*Irg1* mice (*n* = 6 per group). Scale bar = 25 μm. (**G**–**I**) Concentration-response curves for contraction (**G**), endothelium-dependent relaxation (**H**), and endothelium-independent relaxation (**I**) in mesenteric resistance arteries from AAV9-α-Smmhc-null and AAV9-α-Smmhc-*Irg1* mice infused with Ang II for 14 days (*n* = 6 per group). (**J**) Protein levels of COL1A1, α-SMA, and SM22α in aortas from AAV9-α-Smmhc-null and AAV9-α-Smmhc-*Irg1* mice infused with saline or Ang II for 14 days (*n* = 5–6 per group). Data are presented as mean ± SEM. Statistical comparisons were performed using Student’s *t*-test for two groups and two-way ANOVA with appropriate post hoc tests for multiple groups; AUC values for concentration-response curves (**G**–**I**) were compared by unpaired *t*-test. ns, *p* > 0.05; * *p* < 0.05; ** *p* < 0.01; *** *p* < 0.001; **** *p* < 0.0001. Abbreviations: SBP, systolic blood pressure; DBP, diastolic blood pressure; Ang II, angiotensin II; COL1A1, collagen 1α1; α-SMA, α-smooth muscle actin; SM22α, smooth muscle 22α; AUC, area under the log concentration-response curve; SEM, standard error of the mean.

To further investigate the role of the IRG1–ITA axis in hypertension, we generated SMC-specific *Irg1* overexpression mice by tail-vein injection of an AAV9 vector carrying the mouse α-smooth muscle myosin heavy chain (α-Smmhc) promoter (AAV9-α-Smmhc-*Irg1*). Two weeks after injection of AAV9-α-Smmhc-*Irg1*, IRG1 protein expression and ITA levels in mouse aortic tissues were examined. Compared with the AAV9 empty vector control group (AAV9-α-Smmhc-null), both IRG1 protein expression and ITA levels were markedly upregulated in the aortas of AAV9-α-Smmhc-*Irg1*-treated mice (*p* < 0.0001, Supplementary Figure S2C,D). Under physiological conditions, no significant differences in SBP or DBP were observed between the AAV9-α-Smmhc-null and AAV9-α-Smmhc-*Irg1* groups (Figure 3A,B). During Ang II infusion to induce hypertension, mice from AAV9-α-Smmhc-*Irg1* group exhibited significantly higher SBP (tail-cuff: *p* < 0.05; radiotelemetry: *p* < 0.0001) and DBP (radiotelemetry: *p* < 0.0001) (Figure 3A–D), more severe aortic medial thickening (*p* < 0.05) and collagen deposition (*p* < 0.05) (Figure 3E,F), exacerbated endothelium-independent vasodilatory dysfunction (*p* < 0.05, Figure 3G–I and Figure S4A–C), and more pronounced VSMC phenotypic switching (Figure 3J) compared with AAV9-α-Smmhc-null controls, as evidenced by increased COL1A1 (*p* < 0.05), and decreased α-SMA (*p* < 0.01) and SM22α (*p* < 0.05). Collectively, these findings demonstrate that SMC-specific overexpression of IRG1 exacerbates Ang II-induced hypertension and vascular remodeling.

To further investigate the role of ITA, the downstream metabolite of IRG1, in hypertension, we administered exogenous ITA intraperitoneally (75 mg/kg every 2 days) to WT C57BL/6 mice during the two-week Ang II or saline infusion period. Although baseline SBP and DBP were comparable between the saline and ITA groups, exogenous ITA supplementation significantly exacerbated Ang II-induced blood pressure elevation (*p* < 0.05, Supplementary Figure S5A,B), aggravated vascular remodeling (*p* < 0.0001, Supplementary Figure S5C,D), further exacerbated endothelium-independent vasodilatory dysfunction (*p* < 0.05, Supplementary Figure S5E–G), and promoted VSMC phenotypic switching, as evidenced by increased COL1A1 (*p* < 0.05) and decreased α-SMA (*p* < 0.001) and SM22α (*p* < 0.001) (Supplementary Figure S5H). In contrast, endothelium-dependent relaxation (induced by ACh) showed no consistent alteration (Supplementary Figure S5F), indicating that the IRG1–ITA axis predominantly impairs endothelium-independent relaxation rather than directly targeting endothelial cells. These results indicate that exogenous ITA exacerbates a series of vascular pathological changes in hypertension.

### 3.4. Activated IRG1–ITA Axis Aggravates Ang II-Induced HASMC Proliferation, Migration, and Phenotypic Switching

**Figure 4:**
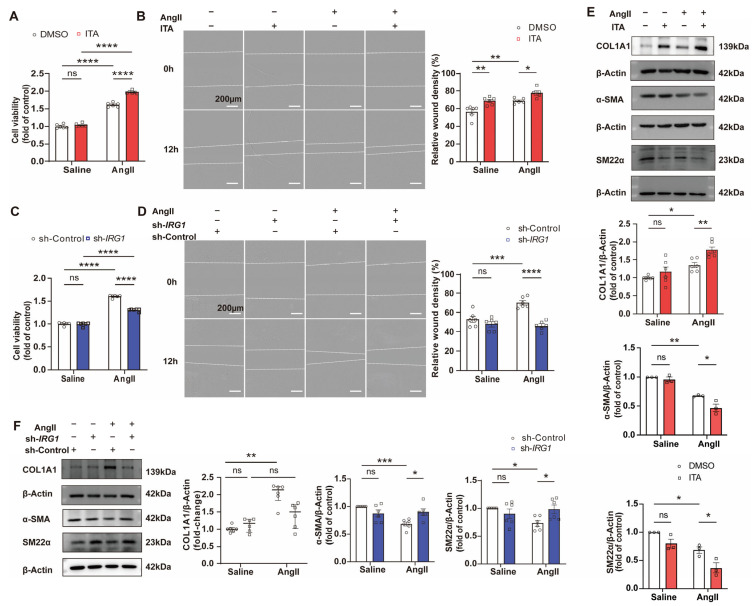
IRG1–ITA promotes Ang II-induced proliferation, migration, and phenotypic switching of HASMCs. (**A**) CCK-8 assay was used to evaluate the effect of ITA on HASMC proliferation with or without Ang II (1 × 10^−6^ M) stimulation (*n* = 6 per group). (**B**) Wound healing assay: representative images of HASMC migration at 0 and 12 h after treatment with ITA and/or Ang II (1 × 10^−6^ M) (*n* = 5–6 per group). Scale bar = 200 μm. (**C**) CCK-8 assay was performed to evaluate the effect of sh-*IRG1* on HASMC proliferation with or without Ang II (1 × 10^−6^ M) stimulation (*n* = 6 per group). (**D**) Representative images of wound healing assay illustrating HASMC migration following transfection with sh-*IRG1* with or without Ang II (1 × 10^−6^ M) at 0 h and 12 h (*n* = 6 per group). Scale bar = 200 μm. (**E**,**F**) Western blot analysis of COL1A1, α-SMA, and SM22α protein expression in HASMCs treated with ITA (**E**) or transfected with sh-*IRG1* (**F**) in the presence or absence of Ang II (1 × 10^−6^ M) for 48 h (*n* = 3–7 per group). For normally distributed data, results are shown as mean ± SEM and analyzed by two-way ANOVA with Tukey’s post hoc test. For non-normally distributed data, results are shown as scatter plots with median (IQR) and analyzed by Kruskal–Wallis test with Dunn’s post hoc test. ns, *p* > 0.05; * *p* < 0.05; ** *p* < 0.01; *** *p* < 0.001; **** *p* < 0.0001. Abbreviations: *sh-IRG1,* short hairpin RNA targeting IRG1; DMSO, dimethyl sulfoxide; ITA, itaconate; COL1A1, collagen 1α1; α-SMA, α-smooth muscle actin; SM22α, smooth muscle 22α; IQR, interquartile range; SEM, standard error of the mean.

Based on the above in vivo findings, we investigated the effects of the IRG1–ITA axis on the proliferation, migration, and phenotypic switching of HASMCs in vitro. Exogenous ITA treatment exacerbated Ang II-induced proliferation (*p* < 0.0001) and migration (*p* < 0.05) of HASMCs (Figure 4A,B), whereas IRG1 knockdown via sh-*IRG1* suppressed these Ang II-induced pathological alterations (*p* < 0.0001, Figure 4C,D). Furthermore, ITA aggravated Ang II-induced phenotypic switching of HASMCs, as evidenced by further upregulation of COL1A1 (*p* < 0.01) and downregulation of α-SMA (*p* < 0.05) and SM22α (*p* < 0.05) (Figure 4E). Conversely, sh-*IRG1* effectively attenuated these Ang II-induced alterations, with significant restoration of α-SMA (*p* < 0.05) and SM22α (*p* < 0.05), together with a downward trend in COL1A1 expression that did not reach statistical significance (Figure 4E,F and Figure S6A).

### 3.5. ITA Binds to CTH at the Cysteine 229 (Cys229) Residue

**Figure 5:**
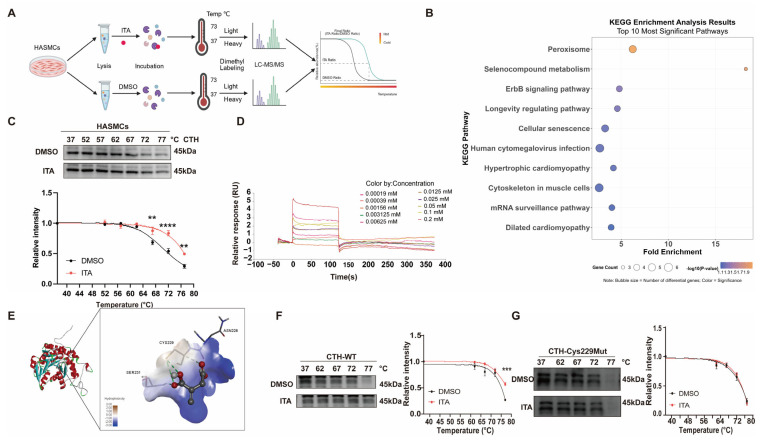
ITA directly interacts with CTH at the Cys229 residue. (**A**) Schematic workflow of TPP for identifying ITA target proteins in HASMCs. Cells were treated with ITA or DMSO, lysed, heated at 37 °C or 73 °C, and then analyzed by LC-MS/MS (*n* = 3 per group). (**B**) Top 10 enriched biological pathways from KEGG enrichment analysis. The analysis was performed on the top 200 proteins with significantly increased thermal stability following ITA treatment, as detected by TPP (ratio ≥ 1.2, *p* < 0.05). (**C**) CETSA analysis of CTH thermal stability in HASMCs treated with or without ITA (*n* = 3 per group). (**D**) SPR analysis of the binding between ITA and purified CTH protein at various concentrations. (**E**) Molecular docking predicting the amino acid residues in CTH that bind to ITA. (**F**,**G**) CETSA analysis of HEK293A cells transfected with CTH-WT (**F**) or CTH-Cys229Mut (**G**). Cells were treated with DMSO or ITA (*n* = 3 per group). Data are presented as mean ± SEM. For CETSA data (**C**,**F**,**G**), two-way ANOVA with treatment and temperature as factors, followed by Bonferroni’s post hoc test, was used for multiple comparisons. ** *p* < 0.01, *** *p* < 0.001, **** *p* < 0.0001. Abbreviations: ITA, itaconate; DMSO, dimethyl sulfoxide; CTH-WT, wild-type cystathionine gamma-lyase; CTH-Cys229Mut, CTH cysteine 229 mutant; HASMC, human aortic smooth muscle cell; SPR, surface plasmon resonance; LC-MS/MS, liquid chromatography-tandem mass spectrometry; KEGG, Kyoto Encyclopedia of Genes and Genomes.

To identify the downstream targets of ITA in VSMCs, we performed TPP in HASMCs (Figure 5A). A total of 5737 proteins were detected, among which 1348 exhibited significantly increased thermal stability after ITA incubation (ratio ≥ 1.2, *p* < 0.05). The top 200 proteins with the lowest *p*-values were selected for pathway enrichment analysis. KEGG pathway analysis identified selenocompound metabolism as the pathway with the highest fold enrichment among the top enriched pathways, which involves three proteins: SEPSECS, SEPHS2, and CTH (Figure 5B). Subsequent analysis further confirmed the significance of these genes, as they were also found to be enriched in the WikiPathways analysis of Selenium metabolism and selenoproteins and GO analysis of protein binding (Supplementary Figure S7A,B). Given the established role of CTH in the development and progression of hypertension, we further investigated whether ITA interacts with CTH. CETSA demonstrated that ITA enhanced the thermal stability of CTH in HASMCs, with significant differences between ITA- and DMSO-treated groups at 67 °C (*p* < 0.01), 72 °C (*p* < 0.0001), and 77 °C (*p* < 0.01) (Figure 5C). SPR analysis further verified a direct and concentration-dependent interaction between ITA and CTH (Kd: 5.34 μM) (Figure 5D). Molecular docking predicted that the interaction between ITA and CTH may involve residues Cys229, Asn228, or Ser231 (Figure 5E). To validate the molecular docking predictions, we utilized HEK293A cells, which exhibited negligible endogenous CTH protein expression. CTH protein expression was markedly upregulated in HEK293A cells successfully transfected with the CTH-WT overexpression plasmid (Supplementary Figure S8A). Subsequently, CETSA was performed on HEK293A cells transfected with CTH-WT or CTH carrying individual site mutations (CTH-Cys229Mut, CTH-Asn228Mut, CTH-Ser231Mut) to validate the binding site of ITA to CTH. The results demonstrated that mutation at Cys229 disrupted the interaction between ITA and CTH (*p* > 0.05), whereas mutation at the other residues had no such effect (Figure 5F,G and Figure S8B,C). These findings identify Cys229 as the major residue mediating the binding between CTH and ITA.

### 3.6. ITA Aggravates Ang II-Induced Downregulation of CTH Expression, Enzymatic Activity, and Endogenous H_2_S Production

**Figure 6:**
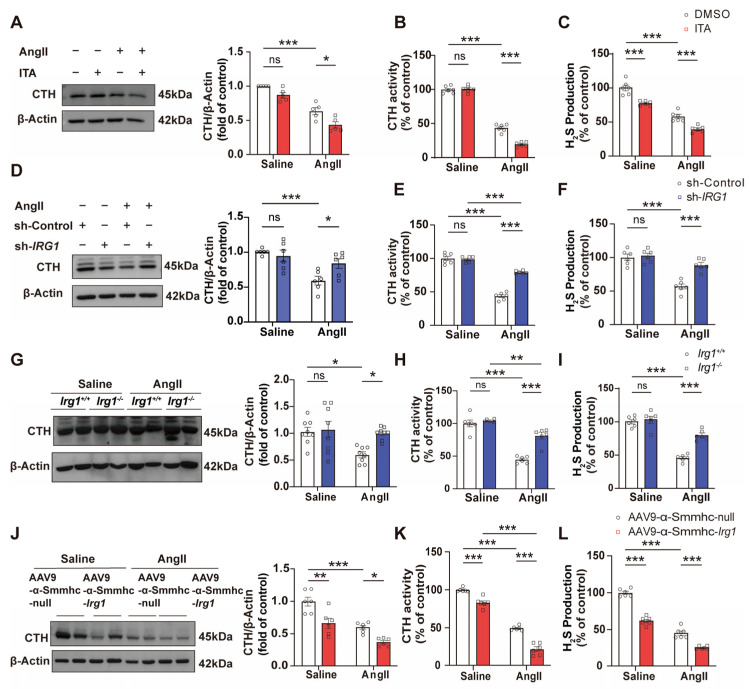
Effects of IRG1–ITA axis on CTH expression, enzymatic activity, and H_2_S production in mouse aortas and HASMCs. (**A**–**C**) Protein expression (**A**), enzymatic activity (**B**), and H_2_S production (**C**) of CTH in HASMCs under four treatment conditions: control, ITA, Ang II, and Ang II + ITA (*n* = 5–6 per group). (**D**–**F**) Protein expression (**D**), enzymatic activity (**E**), and H_2_S production (**F**) of CTH in HASMCs under four treatment conditions: control, sh-*IRG1*, Ang II, and Ang II + sh-*IRG1* (*n* = 6 per group). (**G**–**I**) CTH protein expression (**G**), enzymatic activity (**H**) and H_2_S production (**I**) in aortas from *Irg1^+/+^* and *Irg1^−/−^* mice with or without Ang II infusion (*n* = 6–8 per group). (**J**–**L**) CTH protein expression (**J**), enzymatic activity (**K**) and H_2_S production (**L**) in aortas from AAV9-α-Smmhc-null and AAV9-α-Smmhc-*Irg1* mice with or without Ang II infusion (*n* = 6 per group). Data are presented as mean ± SEM and were analyzed by two-way ANOVA with Tukey’s post hoc test. ns, *p* > 0.05; * *p* < 0.05, ** *p* < 0.01, *** *p* < 0.001. Abbreviations: ITA, itaconate; CTH, cystathionine gamma-lyase; H_2_S, hydrogen sulfide; sh-*IRG1*, short hairpin RNA targeting IRG1; WT, wild-type; SEM, standard error of the mean.

We next investigated the effects of ITA on CTH expression, enzymatic activity, and endogenous H_2_S production. In vitro, ITA aggravated Ang II-induced downregulation of CTH protein levels (*p* < 0.05), enzymatic activity (*p* < 0.001), and H_2_S production (*p* < 0.001) in HASMCs (Figure 6A–C), whereas sh-*IRG1* effectively alleviated Ang II-induced inhibition of CTH protein levels (*p* < 0.05), enzymatic activity (*p* < 0.001), and H_2_S production (*p* < 0.001) in HASMCs (Figure 6D–F). In vivo, *Irg1* knockout attenuated Ang II-induced inhibition of CTH protein levels (*p* < 0.05), enzymatic activity (*p* < 0.001), and H_2_S production (*p* < 0.001) in the aorta (Figure 6G–I). In contrast, SMC-specific *Irg1* overexpression further suppressed CTH expression (*p* < 0.05), enzymatic activity (*p* < 0.001), and H_2_S production (*p* < 0.001) on top of Ang II stimulation (Figure 6J–L). Immunohistochemical staining of aortic sections from SMC-specific *Irg1* overexpression mice further confirmed that CTH protein was predominantly localized in the vascular media and was markedly reduced upon *Irg1* overexpression (*p* < 0.001, Supplementary Figure S9A).

To further exclude potential confounding contributions from other H_2_S-generating enzymes, we examined CBS protein expression in aortic tissues from both *Irg1* knockout and SMC-specific *Irg1* overexpression mice. In aortic tissues, CBS expression was barely detectable and remained unaffected by *Irg1* knockout or SMC-specific *Irg1* overexpression (Supplementary Figure S10A,B), indicating that the IRG1–ITA axis modulates H_2_S production predominantly through CTH. We also measured plasma cysteine levels using a commercial detection kit, as L-cysteine is the predominant form of cysteine in plasma and serves as the substrate for CTH-mediated H_2_S synthesis. Notably, plasma cysteine levels showed an inverse pattern relative to CTH protein expression and enzymatic activity. Under hypertensive conditions, CTH was downregulated and plasma cysteine levels were elevated. In SMC-specific *Irg1* overexpression mice, CTH was further suppressed and plasma cysteine levels were correspondingly increased (*p* < 0.0001, Supplementary Figure S11A). Conversely, in *Irg1* knockout mice, CTH expression and activity were partially restored, accompanied by a significant reversal of elevated plasma cysteine levels (*p* < 0.0001, Supplementary Figure S11B). To confirm the critical role of the Cys229 binding site in ITA-mediated inhibition of CTH, we examined the effect of exogenous ITA on H_2_S levels in HEK293A cells transfected with either CTH-WT or CTH-Cys229Mut plasmids. The results showed that the Cys229 mutation preserved basal H_2_S production and significantly reversed ITA-mediated inhibition of CTH, as evidenced by the restoration of H_2_S levels after ITA treatment (*p* < 0.001, Supplementary Figure S12A). These findings suggest that ITA-mediated inhibition of CTH depends on the Cys229 residue within the protein, ultimately leading to reduced endogenous H_2_S production.

## 4. Discussion

Although accumulating evidence has highlighted the critical role of metabolic reprogramming in hypertension and vascular remodeling, the function of the IRG1–ITA axis in this pathological process has remained undefined. In this study, we provide the first evidence that activation of the IRG1–ITA axis acts as a positive regulator accelerating the progression of hypertension. Our in vivo findings demonstrate that genetic ablation of *Irg1* effectively protects against Ang II-induced hypertension, whereas SMC-specific overexpression of *Irg1* or exogenous ITA administration exacerbates these pathological changes. These observations were further corroborated in vitro, where *IRG1* knockdown attenuated, while exogenous ITA promoted, Ang II-induced VSMC proliferation, migration, and phenotypic switching. Mechanistically, we identified that IRG1-derived ITA functions as an endogenous inhibitor of CTH by directly binding to its Cys229 residue, thereby suppressing H_2_S production in VSMCs. Collectively, these findings not only advance our understanding of vascular metabolic reprogramming but also establish the IRG1–ITA axis as a promising therapeutic target for hypertension.

The IRG1–ITA axis has been extensively studied in CVD, and most studies have reported a protective role. For example, in an Ang II-induced AAA mouse model, the ITA derivative 4-octyl itaconate (4-OI) suppressed AAA formation by inhibiting macrophage-mediated inflammation, whereas *Irg1* knockout mice exhibited significantly enhanced AAA formation compared with their WT counterparts [22]. In a mouse model of ischemia–reperfusion (IR) injury, continuous administration of 4-OI for seven days significantly reduced myocardial damage and promoted angiogenesis, whereas *Irg1* deficiency markedly exacerbated cardiac injury, as evidenced by increased infarct size and more severe fibrosis [44]. Similar cardioprotective functions of ITA have been reported in acute myocardial infarction [45], valvular heart disease [46], and chemotherapy-induced cardiotoxicity [47], largely through the activation of Nrf2 and ATF3 pathways [19,48].

However, the role of this axis in hypertension had remained completely unexplored prior to this study. Given the context-dependent nature of IRG1–ITA signaling, we next examined its function in hypertensive settings. In striking contrast to its protective role in other diseases, our findings reveal that ITA functions as a pathogenic driver in hypertension, aggravating blood pressure elevation and vascular remodeling, whereas IRG1 knockout confers protection against hypertension. Intriguingly, even in atherosclerosis—a vascular disease closely related to hypertension—the function of IRG1–ITA remains controversial. Some studies have reported that *Irg1* deficiency exacerbates atherosclerotic burden, while others have suggested that myeloid-specific *Irg1* deletion enhances plaque stability [23,24]. These discrepancies indicate that the IRG1–ITA axis is highly context-dependent, with its biological outcomes varying according to disease type, cell type, and pathological milieu. In line with this notion, our study uncovers a previously unrecognized deleterious role of this axis specifically within VSMCs during hypertension, thereby significantly extending the current paradigm.

To further validate the pathogenic role of ITA, we employed two complementary approaches: systemic ITA administration and SMC-specific *Irg1* overexpression. Both strategies similarly aggravated Ang II-induced hypertension, vascular remodeling, VSMC phenotypic switching, and vascular dysfunction. This convergence supports a causative role of increased ITA signaling in hypertension. Nonetheless, these approaches differ: systemic ITA exposes multiple tissues to the metabolite, whereas SMC-specific *Irg1* overexpression locally augments ITA production in VSMCs. The similar phenotypes reinforce that ITA acts at least in part through VSMC-autonomous mechanisms, while any divergence may reflect additional functional effects of systemic ITA on extravascular tissues. This possibility, together with the potential contribution of other organs—such as the kidney, liver, spleen, or immune cells—to circulating ITA, warrants further investigation. Thus, considering both tissue and cell type specificity is essential when therapeutically targeting this metabolic pathway.

To elucidate the molecular mechanism underlying the role of ITA in VSMCs, we employed TPP, SPR, and CETSA to identify CTH as a direct downstream target of ITA. CTH is a key enzyme responsible for endogenous H_2_S production and primarily mediates H_2_S synthesis in VSMCs and aortic tissues [49]. Although CBS also contributes to H_2_S generation in some tissues, we found that CBS protein was barely detectable in aortic tissues and remained unaffected by *Irg1* knockout or SMC-specific *Irg1* overexpression. These findings exclude a significant role of CBS in contributing to H_2_S production in mouse aortic tissues under our experimental conditions. This is consistent with our finding that ITA directly binds to and inhibits CTH, indicating that the IRG1–ITA axis predominantly modulates vascular H_2_S production through CTH. H_2_S exerts multiple vasoprotective effects, including promoting vasodilation, inhibiting VSMC proliferation and migration, reducing oxidative stress, and limiting inflammatory responses [50]. Conversely, H_2_S deficiency impairs these protective functions by compromising protein S-sulfhydration, a key post-translational modification that regulates ion channels, redox signaling, and inflammatory pathways [51].

The activity of CTH is subject to regulation at multiple levels, encompassing transcriptional control, protein stability, and post-translational modifications [52]. These modifications include modifications at various cysteine residues (e.g., polysulfidation, S-nitrosation, and S-sulfhydration), as well as acetylation, SUMOylation, and phosphorylation [52]. In contrast to these covalent modifications, we found that ITA regulates CTH expression and function through non-covalent binding. Using molecular docking and CETSA, we demonstrated that ITA non-covalently binds to the Cys229 residue of CTH via hydrogen bonds, leading to reduced CTH activity. SPR analysis further confirmed this direct and reversible interaction between ITA and CTH, consistent with a non-covalent binding mode.

In the absence of Ang II stimulation, neither SMC-specific IRG1 overexpression nor ITA administration alone induced hypertension in mice. This is explained by the reversible nature of ITA-mediated CTH inhibition and the robust H_2_S compensatory capacity under physiological conditions. In healthy mouse vessels, high CTH expression together with alternative H_2_S-generating pathways (CBS and 3-MST) compensates for ITA-induced partial H_2_S reduction, maintaining total H_2_S above the functional threshold [53]. In contrast, under Ang II treatment, CTH expression is downregulated and compensatory pathways become exhausted [54], thereby amplifying the inhibitory effect of ITA on CTH. These observations indicate that ITA does not act as a direct initiator of hypertension, but rather serves as a pathogenic amplifier that depends on the vascular pathological state under hypertensive conditions.

In addition, the NO–cGMP pathway—another major vasodilatory signaling cascade—may interact with H_2_S at multiple levels, including through the sulfhydration of eNOS or soluble guanylate cyclase (sGC) [55,56]. In the present study, we assessed vascular responsiveness to an NO donor (SNP) via endothelium-independent relaxation assays in the presence of L-NAME, which provided functional information on smooth muscle sensitivity to exogenous NO. However, the effects of the IRG1–ITA axis on NO production, eNOS phosphorylation, sGC activity, cGMP levels, and downstream PKG signaling were not directly assessed herein. Therefore, whether the IRG1–ITA pathway directly modulates the NO–cGMP cascade remains to be determined. Future studies are needed to dissect the potential interplay between the IRG1–ITA pathway and the NO-cGMP system [57].

Additionally, although Ang II directly induced IRG1 expression in cultured VSMCs, the magnitude of upregulation was considerably lower than that observed in aortic tissues from Ang II-induced hypertensive mice. This discrepancy suggests that Ang II alone does not fully recapitulate the in vivo response. We hypothesize that other factors within the hypertensive vascular microenvironment—including chronic mechanical stretch, perivascular inflammation, infiltrating immune cells (which themselves express IRG1), and endothelial–VSMC crosstalk—act in synergy with Ang II to collectively mediate IRG1–ITA activation. Thus, the robust upregulation of IRG1 in hypertensive arteries likely reflects the integrated action of multiple hypertension-related stimuli.

The Ang II-induced hypertensive model was selected over the salt-sensitive (SS) rat model or spontaneously hypertensive rat (SHR) model for the following reasons. First, Ang II directly engages signaling pathways governing VSMC proliferation and phenotypic switching, rendering this model particularly suitable for investigating new targets involved in VSMC phenotypic switching [58]. In contrast, the SS rats primarily drive hypertension through volume expansion, and the SHR model is complicated by an undefined genetic background [59,60]. Second, the lack of a suitable rat IRG1 antibody has limited our ability to conduct studies in SS rats or the SHR model, further justifying our exclusive use of the Ang II model in this study. Nevertheless, despite our in vitro and SPR data showing that ITA directly binds to CTH independently of Ang II stimulation, whether activation of the IRG1–ITA axis is a general phenomenon in hypertension or is specifically associated with Ang II-induced hypertension remains to be determined. Future studies using additional hypertensive models, including SS rats and SHR, are needed to determine the broader relevance of this regulatory axis.

It is well established that the kidney plays a particularly critical role in long-term blood pressure control through the regulation of sodium and water balance. However, in the present model of Ang II-induced hypertension over a 14-day period, the primary pathological changes occur within the vasculature, whereas renal sodium handling typically becomes more prominent in long-term or volume-dependent hypertensive settings. Therefore, our experimental focus was placed on vascular mechanisms rather than renal function. Accordingly, we did not directly assess renal function, renal IRG1/ITA expression, urinary sodium excretion, or tubular transporter expression, although our findings do not exclude potential renal contributions [61]. Whether the IRG1–ITA axis also participates in regulating renal tubular Na^+^ handling or renal function in hypertension remains an open question that warrants further investigation.

Despite the important findings of this study, several limitations should be acknowledged: First, lack of clinical validation. All of the present findings are derived from animal models and in vitro experiments. Future studies are required to validate changes in IRG1 and ITA levels in aortic tissues or plasma from hypertensive patients and to explore their correlation with blood pressure levels or the degree of vascular stiffness, thereby enhancing the translational value of our findings. Second, incomplete causal validation. This study did not perform rescue experiments, such as H_2_S donor supplementation in *Irg1*-overexpressing mice. Future functional rescue experiments are needed to further consolidate the causal chain from the IRG1–ITA axis to the CTH/H_2_S pathway. Third, the use of global *Irg1* knockout mice in this study does not exclude potential contributions from other cell types, and the AAV9-mediated SMC-specific overexpression may still exert off-target effects in non-vascular tissues. Future generation of SMC-specific *Irg1* knockout mice using CRISPR/Cas9 technology will therefore be required to more precisely validate the independent role of this axis in VSMCs. Fourth, ambulatory 24 h blood pressure was measured at only a single time point (day 14) after Ang II treatment, and the sample size was limited. Nevertheless, the consistency with tail-cuff measurements strengthens the credibility of our conclusions. Fifth, all in vivo experiments were performed solely in male mice in order to reduce experimental variability due to hormonal fluctuations. Thus, the present findings regarding the IRG1–ITA/CTH/H_2_S axis in hypertension should be interpreted with caution when extrapolated to females. Future studies incorporating female mice across different hormonal statuses are needed to clarify whether the alterations in this metabolic pathway exhibit sex-dependent patterns during hypertension.

In conclusion, this study reveals a critical regulatory role of the IRG1–ITA axis in hypertension, with the CTH/H_2_S pathway serving as a downstream effector regulating blood pressure and vascular remodeling. Hypertensive stimuli upregulate IRG1 and ITA expression in VSMCs, where ITA binds to and inhibits CTH enzymatic activity through its Cys229 residue, leading to reduced H_2_S levels and ultimately promoting blood pressure elevation and vascular remodeling. Targeting the IRG1–ITA axis in VSMCs represents a promising therapeutic strategy for Ang II-induced hypertension.

## Data Availability

The original contributions presented in this study are included in the article/Supplementary Material. Further inquiries can be directed to the corresponding author Jian Liu. The raw data supporting the conclusions of this article will be made available by the authors on request.

## Supplementary Materials

The following supporting information can be downloaded at: https://www.mdpi.com/article/10.3390/jcdd13070338/s1: File S1: Supplementary figures (including Figures S1–S12): Figure S1. IRG1 is not induced in endothelial cells but is upregulated in VSMCs under hypertensive conditions or Ang II stimulation. (A) Representative Western blots and quantitative analysis of IRG1 protein in HUVECs treated with Ang II for 0, 24, and 48 h (*n* = 5–6). (B–D) Analysis of *Irg1/IRG1* mRNA expression in endothelial cells (B,C) and VSMCs (D) under hypertensive conditions or Ang II stimulation, using public GEO datasets GSE302827 (B, *n* = 3 per group), GSE211978 (C, *n* = 6 per group), and GSE291516 (D, *n* = 3 or 6 per group). Data are presented as the mean ± SEM. Statistical comparisons were performed using two-tailed Student *t*-test for comparisons between two groups, and one-way ANOVA for comparisons among three groups. ns, *p* > 0.05. Abbreviations: IRG1/*Irg1*, immunoresponsive gene 1; GAPDH, glyceraldehyde-3-phosphate dehydrogenase; EC, endothelial cell; VSMC, vascular smooth muscle cell; HUVEC, human umbilical vein endothelial cell; WKY, Wistar-Kyoto; SHR, spontaneously hypertensive rat. Figure S2. Validation of *Irg1* knockout and overexpression efficiency in mouse aortas. (A) Representative Western blot images and statistical analysis of IRG1 expression in aortas from *Irg1^+/+^* and *Irg1^−/−^* mice with or without Ang II infusion for 14 days (*n* = 6 per group). (B) ITA levels in mouse aortas were measured using an ITA ELISA kit (*n* = 6 per group). (C) Representative Western blot images and statistical analysis of IRG1 expression in aortas from AAV9-α-Smmhc-null and AAV9-α-Smmhc-*Irg1* mice with or without Ang II infusion for 14 days (*n* = 3 per group). (D) ITA levels in mouse aortas were measured using an ITA ELISA kit (*n* = 6 per group). Data are presented as the mean ± SEM. Data were analyzed using two-tailed Student *t*-test for comparisons between two groups, and two-way ANOVA followed by Tukey’s multiple comparisons test for comparisons among three or more groups. ns, *p* > 0.05; ** *p* < 0.01; **** *p* < 0.0001. Abbreviations: IRG1/Irg1: immunoresponsive gene 1; ITA: itaconate; AAV9: adeno-associated virus serotype 9; α-Smmhc: alpha-smooth muscle myosin heavy chain; ELISA, enzyme-linked immunosorbent assay; SEM, standard error of the mean. Figure S3. *Irg1* knockout does not affect resistance artery function in normotensive mice. (A–C) Concentration-response curves for contraction (A), endothelium-dependent relaxation (B), and endothelium-independent relaxation (C) in mesenteric resistance arteries from *Irg1^+/+^* and *Irg1^−/−^* mice under basal conditions (*n* = 6 per group). Data are presented as the mean ± SEM. For the vascular ring concentration-response curves in panels A–C, the trapezoidal rule was used to compute the AUC of each arterial segment, and the resulting AUC values were compared between the two groups using unpaired Student’s *t*-test. ns, *p* > 0.05. Abbreviations: Phe, phenylephrine; ACh, acetylcholine; L-NAME, Nω-nitro-L-arginine methyl ester; SNP, sodium nitroprusside; *Irg1^+/+^, Irg1* WT; *Irg1^−/−^, Irg1* knockout; AUC, area under the curve; SEM, standard error of the mean. Figure S4. SMC-specific *Irg1* overexpression does not affect resistance artery function in normotensive mice. (A–C) Concentration-response curves for contraction (A), endothelium-dependent relaxation (B), and endothelium-independent relaxation (C) in mesenteric resistance arteries from AAV9-α-Smmhc-null and AAV9-α-Smmhc-*Irg1* mice under basal conditions (*n* = 6 per group). Data are presented as the mean ± SEM. For the vascular ring concentration-response curves in panels A–C, the trapezoidal rule was used to compute the AUC of each arterial segment, and the resulting AUC values were compared between the two groups using unpaired Student’s *t*-test. ns, *p* > 0.05. Abbreviations: Phe: phenylephrine; ACh: acetylcholine; L-NAME: Nω-nitro-L-arginine methyl ester; SNP: sodium nitroprusside; AAV9: adeno-associated virus serotype 9; α-Smmhc: alpha-smooth muscle myosin heavy chain; *Irg1*: immunoresponsive gene 1; AUC, area under the curve; SEM, standard error of the mean. Figure S5. Intraperitoneal injection of ITA exacerbates elevated blood pressure, vascular remodeling, and vascular dysfunction in hypertensive mice. (A,B) SBP (A) and DBP (B) in saline-treated and ITA-treated mice infused with or without Ang II for 14 days (*n* = 6–10). (C,D) Representative H&E (C) and Masson’s trichrome staining (D) images, and statistical analysis of aortic media thickness and collagen deposition in thoracic aortas from control mice and ITA-treated mice treated with saline or Ang II for 14 days (*n* = 6). (E–G) Concentration-response curves of contraction (E), endothelium-dependent relaxation (F), and endothelium-independent relaxation (G) in mesenteric arteries from vehicle control mice and ITA-treated mice infused with Ang II for 14 days (*n* = 6). (H) Expression levels of COL1A1, α-SMA, and SM22α in aortas from mice treated with or without ITA and/or Ang II for 14 days (*n* = 5–6). Data are presented as mean ± SEM. Comparisons between two groups were analyzed by two-tailed Student’s *t*-test. Multiple comparisons among groups were performed by two-way ANOVA followed by Tukey’s post hoc test. AUC values for concentration-response curves (E–G) were compared by unpaired Student’s *t*-test. ns, *p* > 0.05; * *p* < 0.05; ** *p* < 0.01; *** *p* < 0.001; **** *p* < 0.0001. Abbreviations: SBP: systolic blood pressure; DBP: diastolic blood pressure; Ang II: angiotensin II; Phe: phenylephrine; ACh: acetylcholine; L-NAME: Nω-nitro-L-arginine methyl ester; SNP: sodium nitroprusside; COL1A1: collagen 1α1; α-SMA: α-smooth muscle actin; SM22α: smooth muscle 22 alpha; ITA: itaconate; AUC, area under the curve; SEM, standard error of the mean. Figure S6. Validation of sh-*IRG1* knockdown efficiency in HASMCs. (A) HASMCs were transfected with sh-Control or sh-*IRG1*, then stimulated with Ang II (1×10^-6^ M) for 48 h. Western blotting showed that IRG1 protein level was markedly decreased in the sh-*IRG1* group under Ang II treatment, indicating successful knockdown of IRG1. β-Actin was used as a loading control. Abbreviations: IRG1, immunoresponsive gene 1; HASMC, human aortic smooth muscle cell; Ang II, angiotensin II; sh-*IRG1*, short hairpin RNA targeting IRG1; SEM, standard error of the mean. Figure S7. Pathway enrichment analysis of TPP. (A,B) Top 10 enriched GO (A) and WikiPathways (B) terms for the top 200 differential proteins with significantly increased thermal stability following ITA treatment (ratio ≥ 1.2, *p* < 0.05), as identified by TPP analysis in HASMCs. Abbreviations: TPP, thermal proteomic profiling; ITA, itaconate; GO, Gene Ontology; HASMC, human aortic smooth muscle cell. Figure S8. Following validation of CTH overexpression plasmid transfection in HEK293A cells, CETSA was performed to analyze ITA binding to site-mutated CTH. (A) Validation of successful transfection of CTH overexpression plasmid in HEK293A cells. (B) CETSA assay was performed to detect the binding between ITA and CTH after transfection of CTH-ASN228 site mutant plasmid into HEK293A cells (*n* = 3). (C) CETSA assay was performed to detect the binding between ITA and CTH after transfection of CTH-SER231 site mutant plasmid into HEK293A cells (*n* = 3). Comparisons between two groups were analyzed by unpaired Student’s *t*-test. Data are presented as mean ± SEM. **** *p* < 0.0001. Abbreviations: ITA, itaconate; CTH-SER231, Cystathionine gamma-lyase Serine 231 mutant; CTH-ASN228Mut, Cystathionine gamma-lyase Asparagine 228 mutant. Figure S9. SMC-specific *Irg1* overexpression aggravates Ang II-induced downregulation of aortic CTH protein. (A) Representative immunohistochemistry micrographs and quantitative mean optical density analysis of CTH in mouse aortas transduced with AAV9-α-SMMHC-null or AAV9-α-SMMHC-*Irg1*, followed by saline or Ang II infusion. Brown signals indicate CTH immunoreactivity; nuclei are stained blue. Scale bar, 50 μm. Quantification data are normalized to saline-treated AAV9-α-SMMHC-null controls. ns, *p* > 0.05; ** *p* < 0.01; *** *p* < 0.001., *n* = 5 per group. Abbreviations: AAV9, adeno-associated virus serotype 9; α-SMMHC, α-smooth muscle myosin heavy chain; Ang II, angiotensin II; CTH, cystathionine gamma-lyase; *Irg1*, immune responsive gene 1; SMC, smooth muscle cell. Figure S10. CBS expression in mouse aorta is not regulated by IRG1. (A) Western blot and quantitative densitometry of CBS in aorta from mice with SMC-specific *Irg1* overexpression (AAV9-α-Smmhc-*Irg1*) or empty vector control (AAV9-α-Smmhc-null) under saline or Ang II treatment (*n* = 5–6). GAPDH was used as loading control. (B) Western blot and quantitative densitometry of CBS in aorta from *Irg1^+/+^* and *Irg1^−/−^* mice with saline or Ang II infusion (*n* = 6). Data are presented as mean ± SEM. Multiple comparisons among groups were performed by two-way ANOVA followed by Tukey’s post-hoc test. ns, *p* > 0.05; * *p* < 0.05; ** *p* < 0.01. Abbreviations: CBS, cystathionine β-synthase; GAPDH, glyceraldehyde-3-phosphate dehydrogenase; Ang II, angiotensin II; AAV9, adeno-associated virus serotype 9; α-SMMHC, α-smooth muscle myosin heavy chain; *Irg1^+/+^, Irg1* WT; *Irg1^−/−^, Irg1* knockout. Figure S11. Circulating Cysteine levels are regulated by IRG1 during Ang II-induced hypertension. (A) Plasma L-cysteine concentrations in mice with SMC-specific *Irg1* overexpression (AAV9-α-Smmhc-*Irg1*) or empty vector control under saline or Ang II infusion (*n* = 6). (B) Plasma cysteine quantification in *Irg1^+/+^* and *Irg1^−/−^* mice with saline or Ang II treatment (*n* = 6). Basal cysteine levels were unchanged between genotypes (ns). Data are shown as mean ± SEM. Statistical analysis was conducted via two-way ANOVA followed by Tukey’s post-hoc test for all pairwise comparisons. ns, *p* > 0.05; *** *p* < 0.001; **** *p* < 0.0001. Figure S12. ITA inhibits CTH-mediated H_2_S production by targeting the Cys229 residue. (A) H_2_S production in HEK293A cells expressing CTH-WT or CTH-229Mut treated with DMSO or ITA. ITA significantly inhibited H_2_S production only in cells expressing CTH-WT, whereas the C229 mutation abrogated this effect (*n* = 6). Data are presented as mean ± SEM. Multiple comparisons among groups were performed by two-way ANOVA followed by Tukey’s multiple comparisons test. ns, *p* > 0.05; *** *p* < 0.001. Abbreviations: H_2_S, hydrogen sulfide; ITA, itaconate; CTH-WT: wild-type cystathionine gamma-lyase; CTH-Cys229Mut: CTH cysteine 229 mutant. File S2: Supplementary Table S1: Group allocation and sample sizes for *in vivo* experiments.

## Abbreviations

The following abbreviations are used in this manuscript:

IRG1	Immunoresponsive gene 1
ITA	Itaconate
CTH	Cystathionine gamma-lyase
H_2_S	Hydrogen sulfide
SMC	Smooth muscle cell
HASMCs	Human aortic smooth muscle cells
VSMCs	Vascular smooth muscle cells
Ang II	Angiotensin II
CCK-8	Cell Counting Kit-8
TPP	Thermal proteomic profiling
CETSA	Cellular thermal shift assay
SPR	Surface plasmon resonance
Cys229	Cysteine 229
WT	Wild-type
Mut	Mutant
SEM	Standard error of the mean
AUC	Area under the log concentration-response curve
CVD	Cardiovascular disease
CBD	Cerebrovascular disease
AAA	Abdominal aortic aneurysm
IQR	Interquartile range
