# Supplementary material for: Immunoresponsive Gene 1–Itaconate Exacerbates Hypertension by Inhibiting the Cystathionine Gamma-Lyase/Hydrogen Sulfide Pathway
Source: J Cardiovasc Dev Dis. 2026 Jul 20;13(7):338. doi: 10.3390/jcdd13070338 (PMC13410097; doi:10.3390/jcdd13070338)

Raw blots

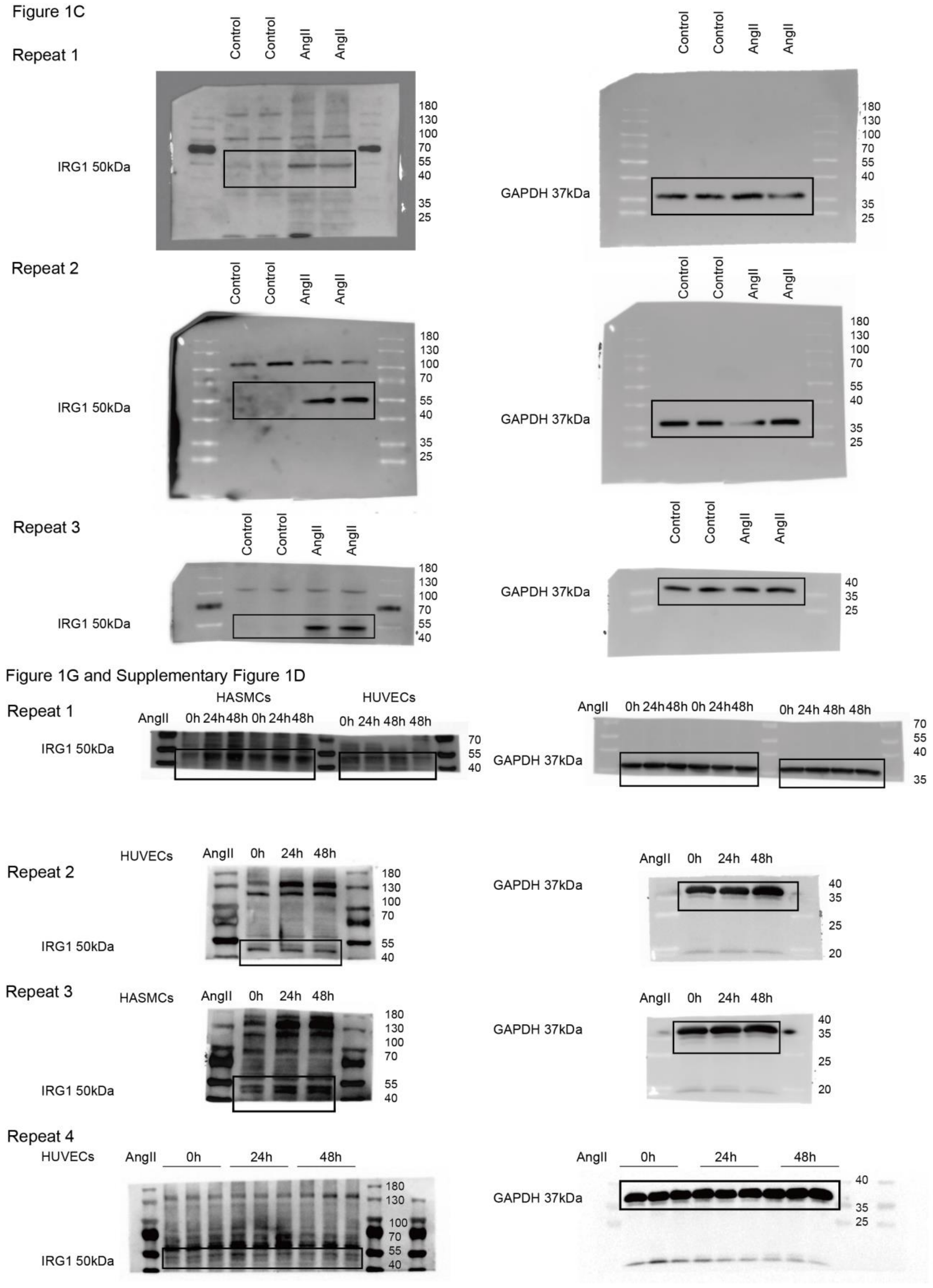
Figure 1C
Repeat 1
Control Control AngII AngII
IRG1 50kDa
180 130 100 70 55 40 35 25
GAPDH 37kDa
Repeat 2
Control Control AngII AngII
IRG1 50kDa
GAPDH 37kDa
Repeat 3
Control Control AngII AngII
IRG1 50kDa
GAPDH 37kDa
Figure 1G and Supplementary Figure 1D
Repeat 1
HASMCs HUVECs
AngII 0h 24h 48h 0h 24h 48h 0h 24h 48h 48h
IRG1 50kDa
GAPDH 37kDa
Repeat 2
HUVECs AngII 0h 24h 48h
IRG1 50kDa
GAPDH 37kDa
Repeat 3
HASMCs AngII 0h 24h 48h
IRG1 50kDa
GAPDH 37kDa
Repeat 4
HUVECs AngII 0h 24h 48h
IRG1 50kDa
GAPDH 37kDa

Figure 2J

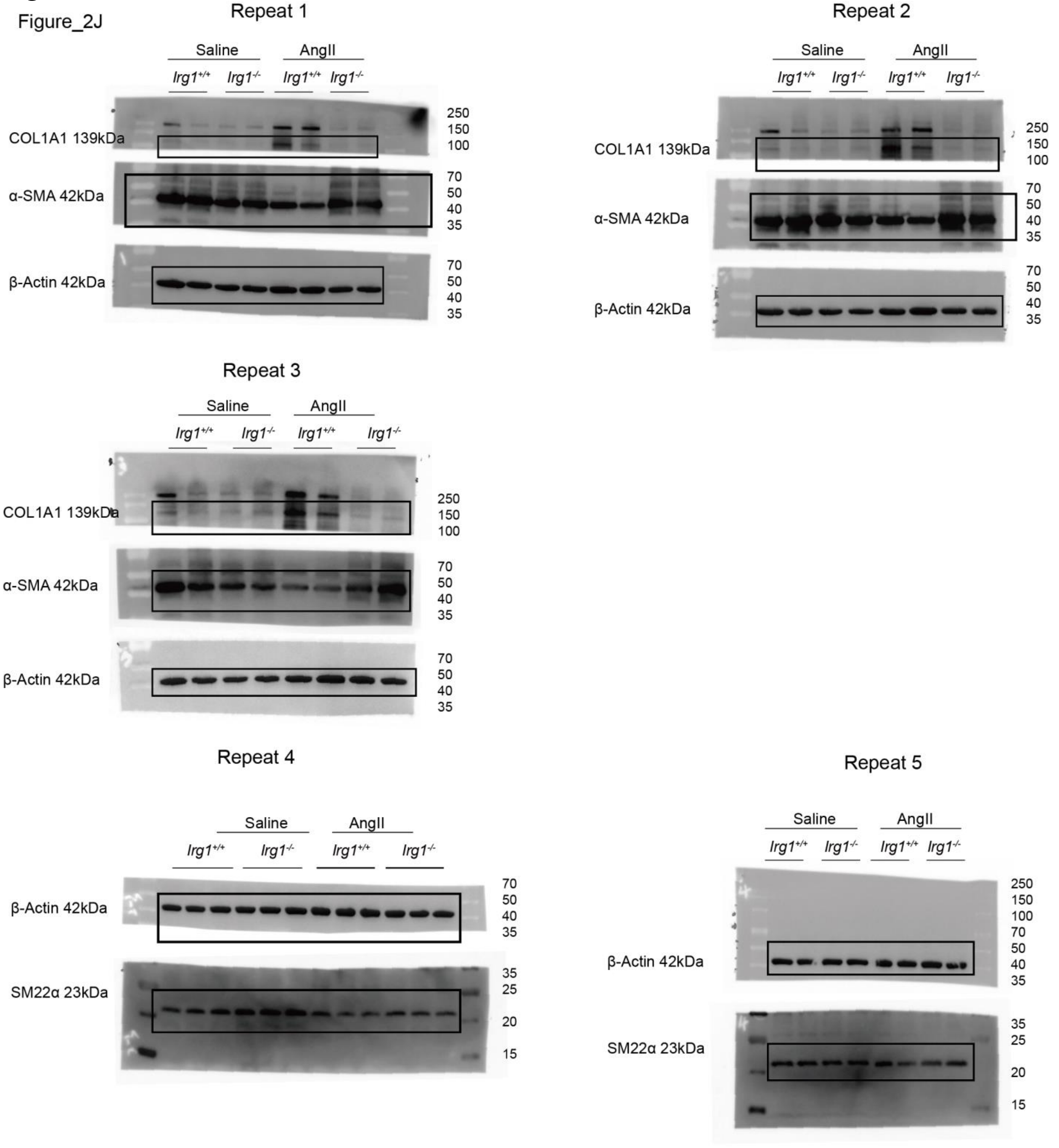
Figure_2J
Repeat 1
Saline
AngII
Irg1+/+
Irg1-/-
Irg1+/+
Irg1-/-
COL1A1 139kDa
α-SMA 42kDa
β-Actin 42kDa
250
150
100
70
50
40
35
Repeat 2
Saline
AngII
Irg1+/+
Irg1-/-
Irg1+/+
Irg1-/-
COL1A1 139kDa
α-SMA 42kDa
β-Actin 42kDa
Repeat 3
Saline
AngII
Irg1+/+
Irg1-/-
Irg1+/+
Irg1-/-
COL1A1 139kDa
α-SMA 42kDa
β-Actin 42kDa
Repeat 4
Saline
AngII
Irg1+/+
Irg1-/-
Irg1+/+
Irg1-/-
β-Actin 42kDa
SM22α 23kDa
25
20
15
Repeat 5
Saline
AngII
Irg1+/+
Irg1-/-
Irg1+/+
Irg1-/-
β-Actin 42kDa
SM22α 23kDa

Figure 3J

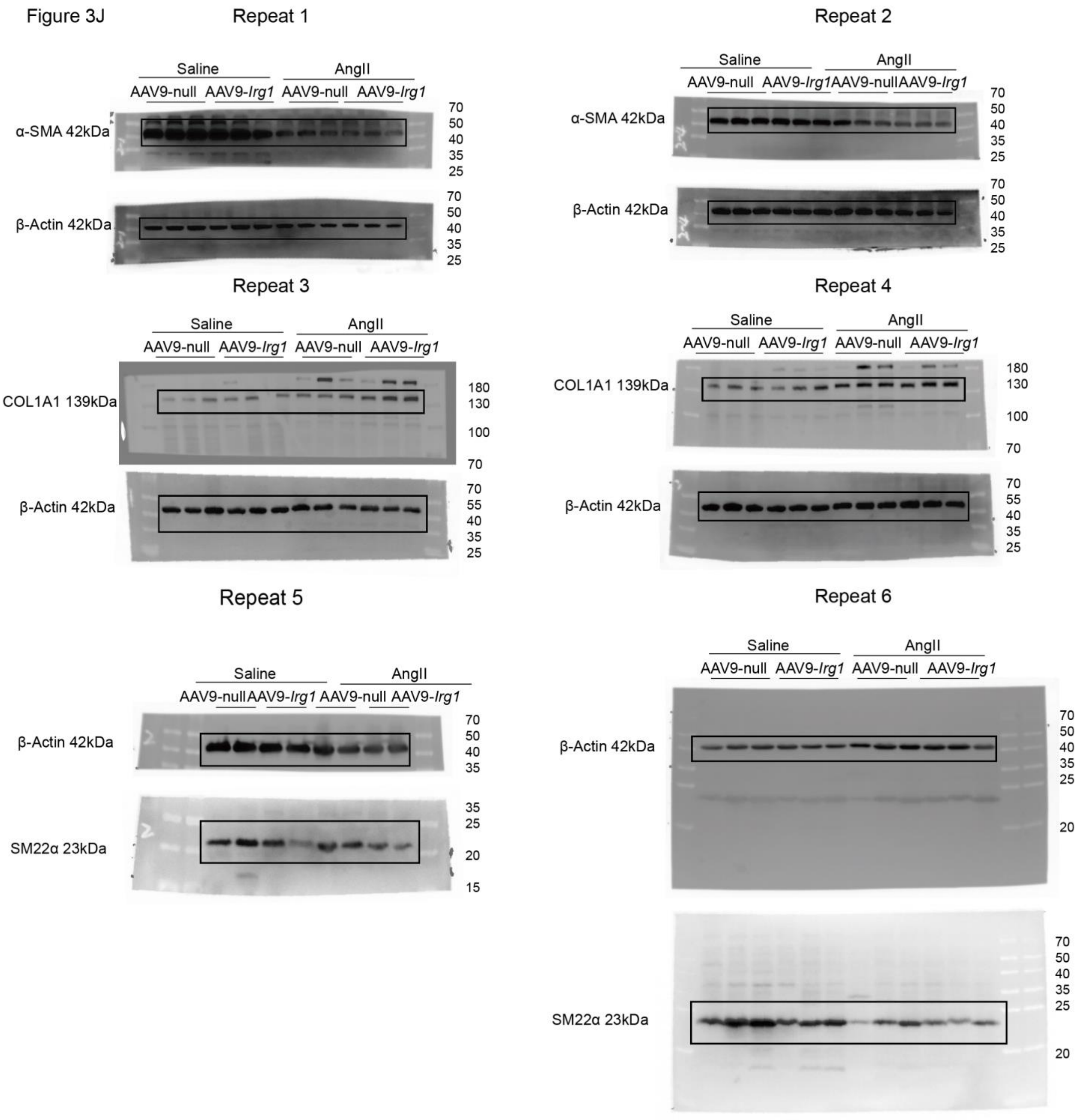

Note:AAV9-null represent AAV9-α-Smmhc-null;AAV9-*Irg1* represent AAV9-α-Smmhc-*Irg1*.

Figure 4E

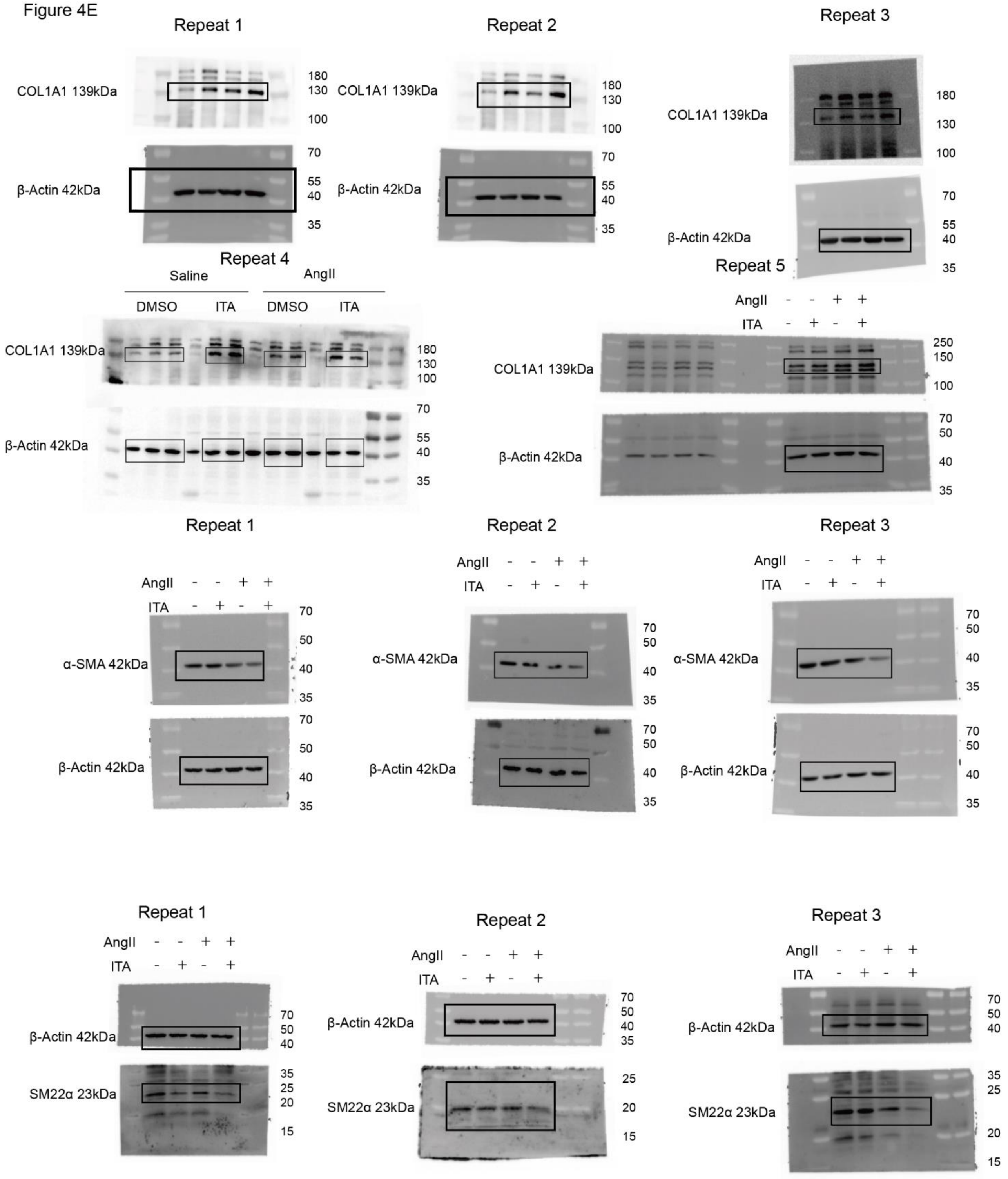

Repeat 1
COL1A1 139kDa
180
130
100
β-Actin 42kDa
70
55
40
35
Repeat 2
COL1A1 139kDa
180
130
100
β-Actin 42kDa
70
55
40
35
Repeat 3
COL1A1 139kDa
180
130
100
β-Actin 42kDa
70
55
40
35
Repeat 4
Saline
AngII
DMSO
ITA
DMSO
ITA
COL1A1 139kDa
180
130
100
β-Actin 42kDa
70
55
40
35
Repeat 5
AngII - - + +
ITA - + - +
COL1A1 139kDa
250
150
100
β-Actin 42kDa
70
50
40
35
Repeat 1
AngII - - + +
ITA - + - +
α-SMA 42kDa
70
50
40
35
β-Actin 42kDa
70
50
40
35
Repeat 2
AngII - - + +
ITA - + - +
α-SMA 42kDa
70
50
40
35
β-Actin 42kDa
70
50
40
35
Repeat 3
AngII - - + +
ITA - + - +
α-SMA 42kDa
70
50
40
35
β-Actin 42kDa
70
50
40
35
Repeat 1
AngII - - + +
ITA - + - +
β-Actin 42kDa
70
50
40
SM22α 23kDa
35
25
20
15
Repeat 2
AngII - - + +
ITA - + - +
β-Actin 42kDa
70
50
40
35
SM22α 23kDa
25
20
15
Repeat 3
AngII - - + +
ITA - + - +
β-Actin 42kDa
70
50
40
SM22α 23kDa
35
25
20
15

Figure_4F and 6D

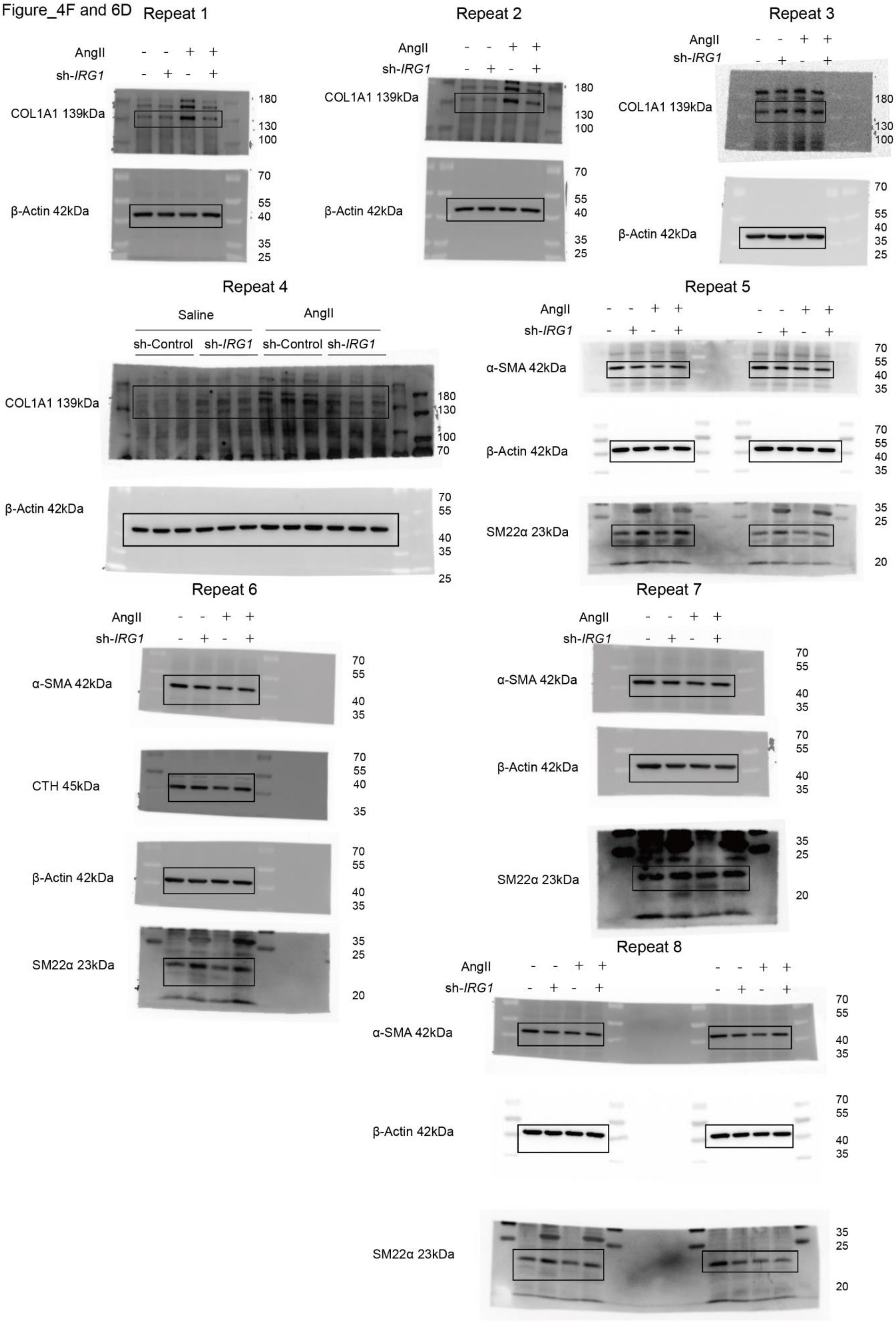

Figure 5C

Repeat 1

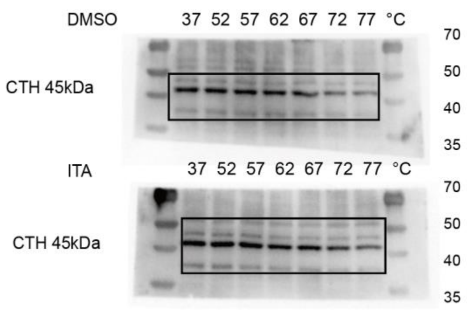

Repeat 2

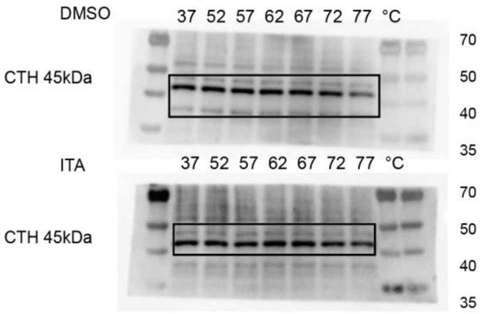

Repeat 3

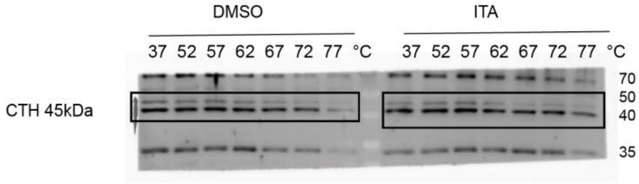

Figure 5F

Repeat 1

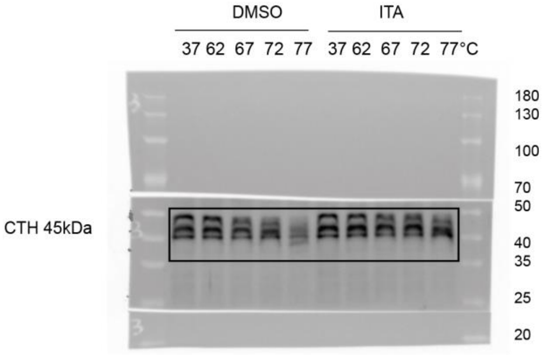

Repeat 2

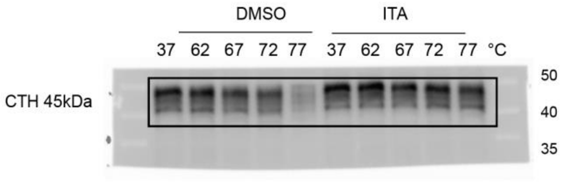

Repeat 3

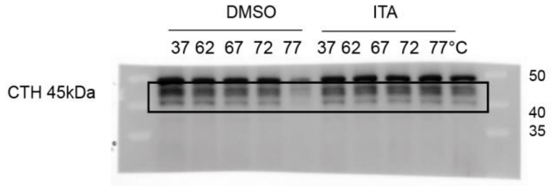

Figure 5G

Repeat 1

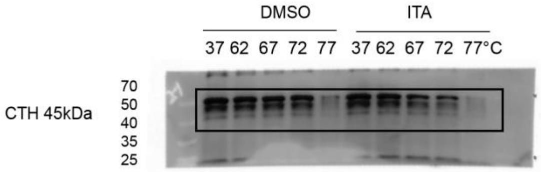

Repeat 2

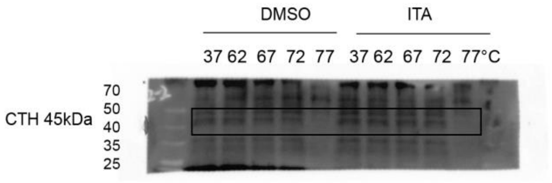

Repeat 3

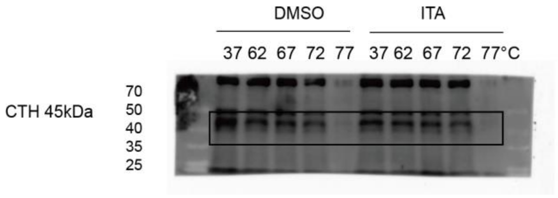

Figure 6A

Repeat 1

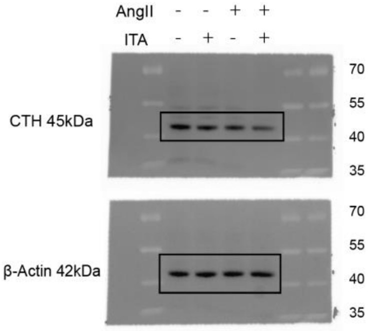

Repeat 2

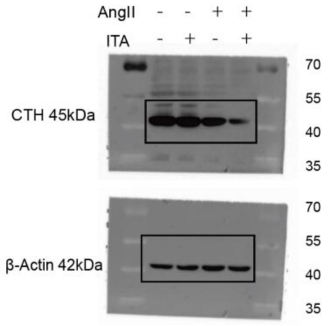

Repeat 3

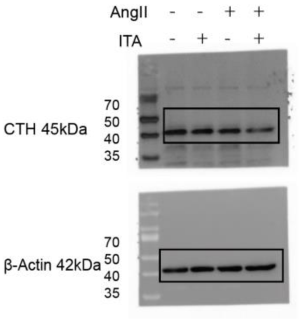

Repeat 4

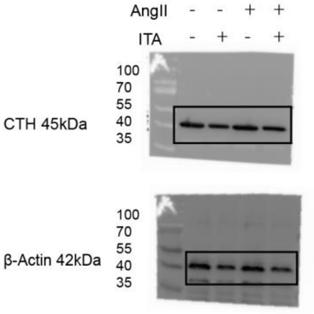

Repeat 5

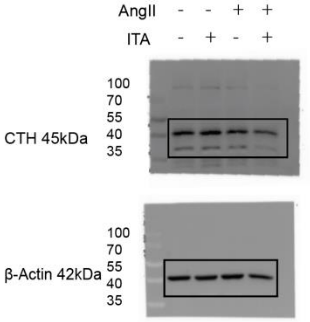

Figure 6D

Repeat 1

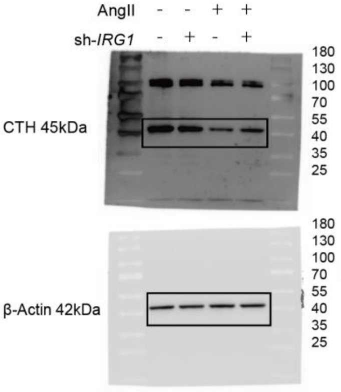

Repeat 2

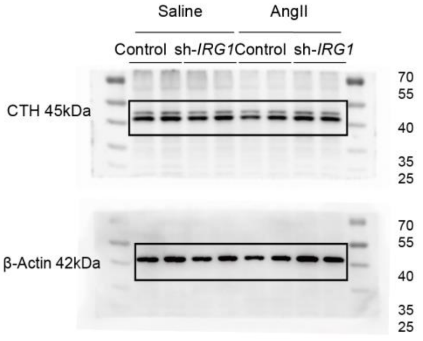

Repeat 3

As shown in Figure 4F
and 6D
Repeat 6

Repeat 4

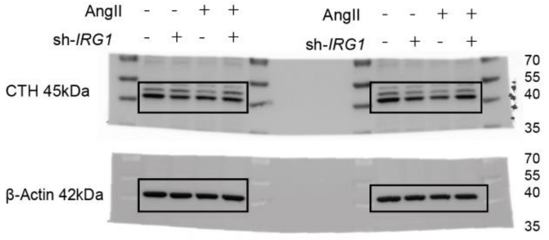

Figure 6G

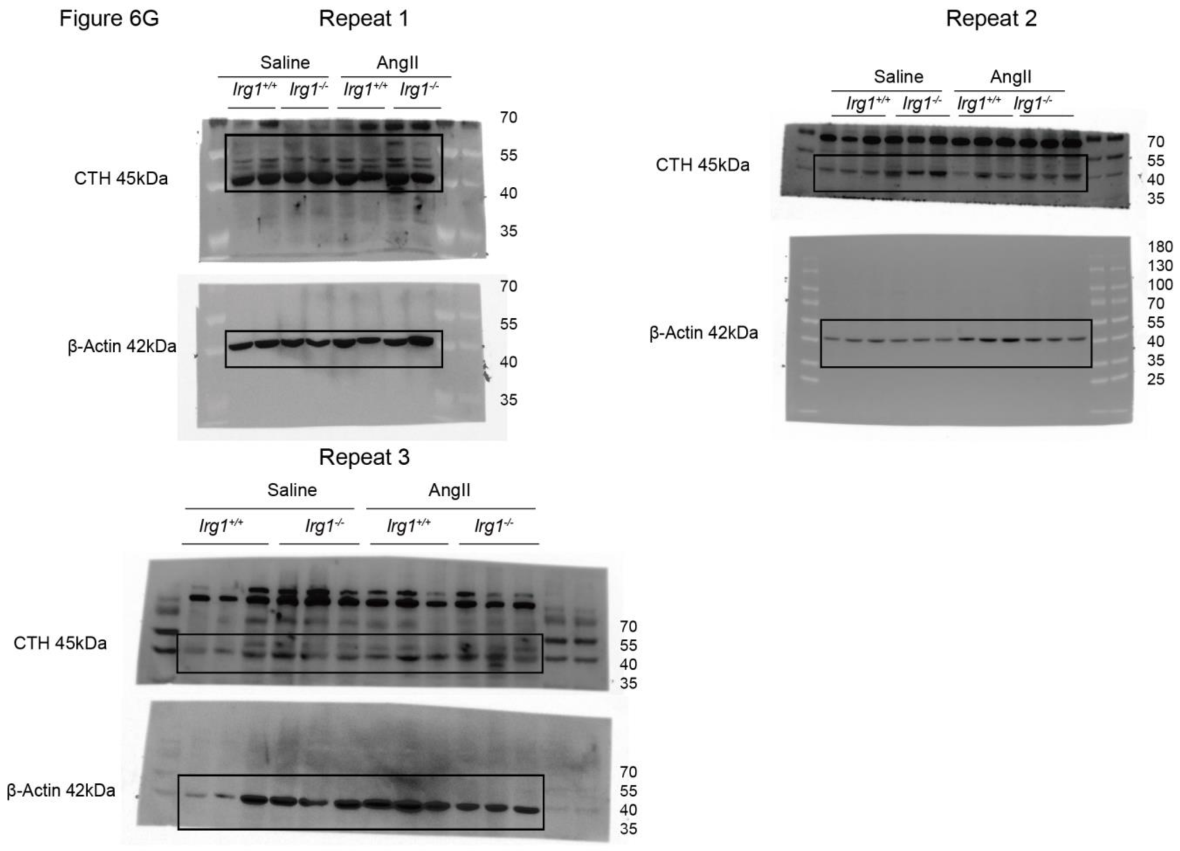

Figure 6J

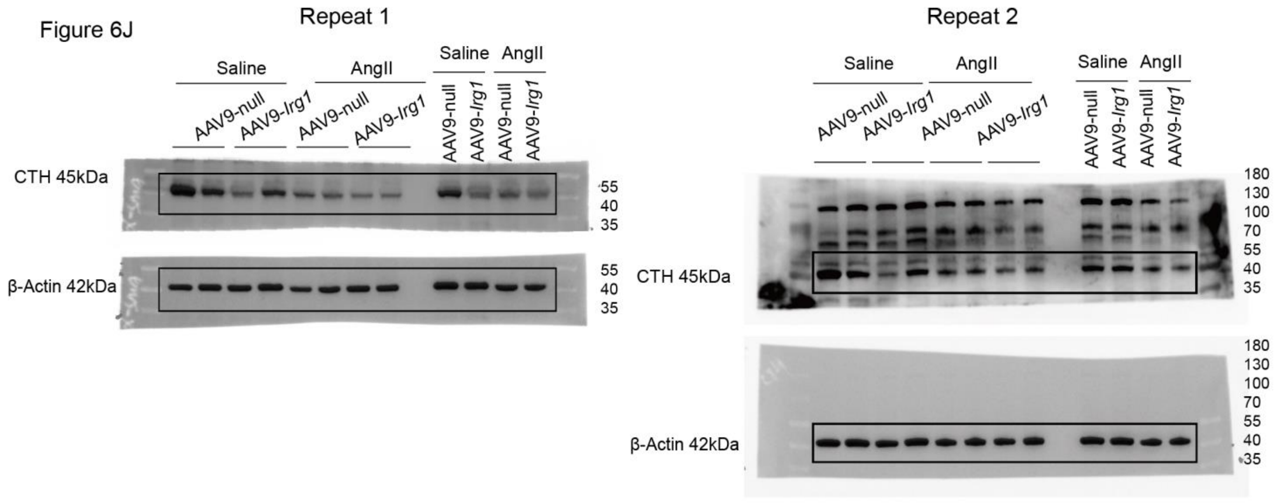

Supplementary Figure 2A

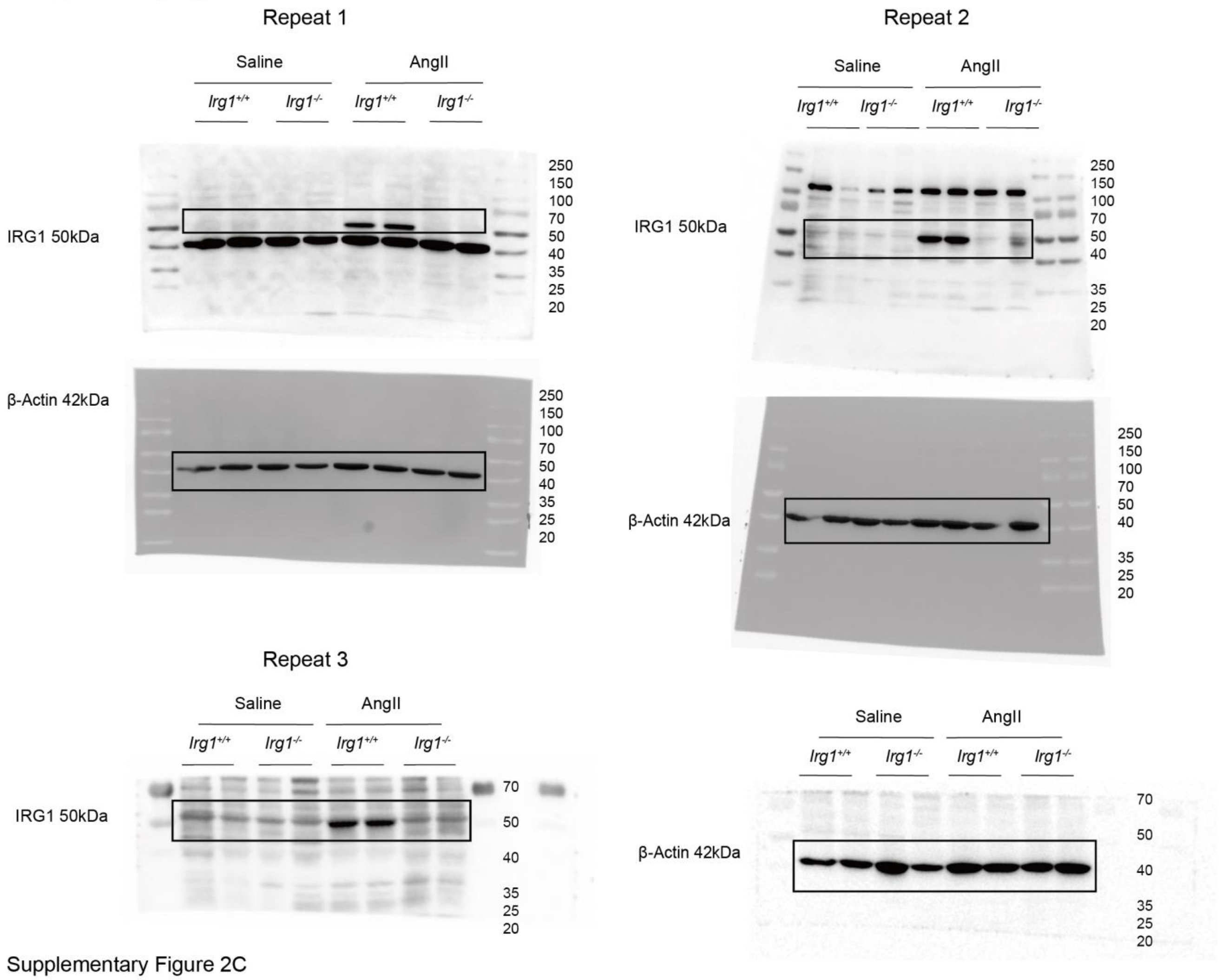

Supplementary Figure 2C

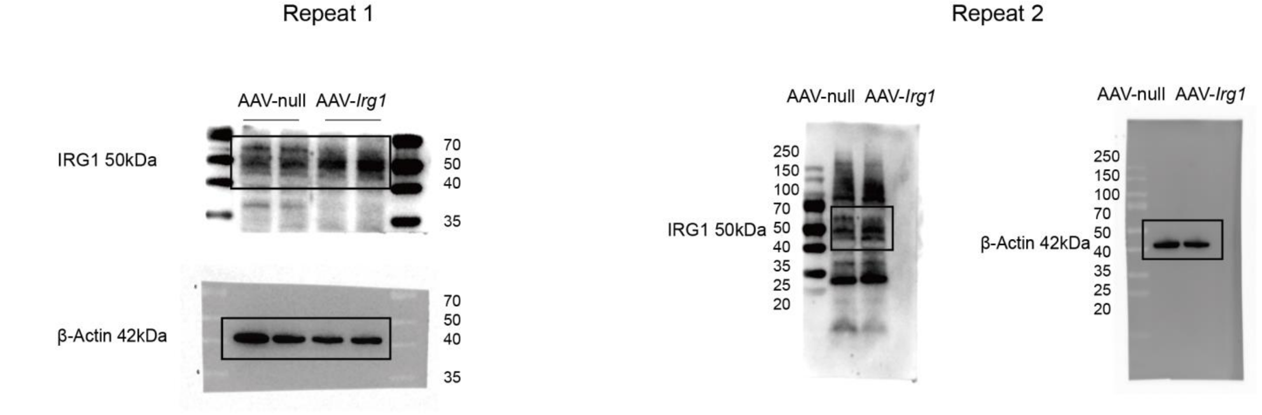

Supplementary Figure_5H

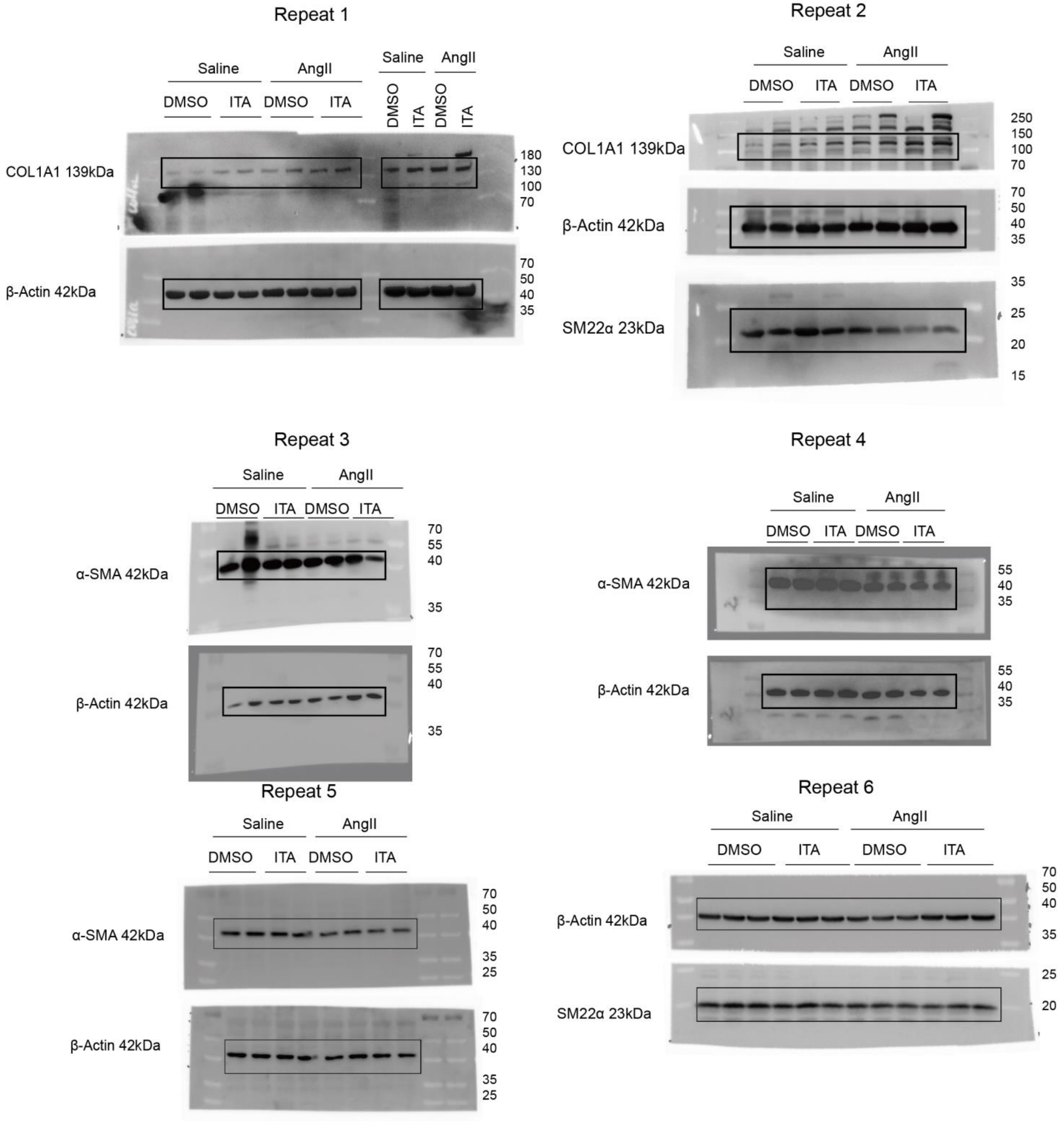

Supplememtary Figure 6

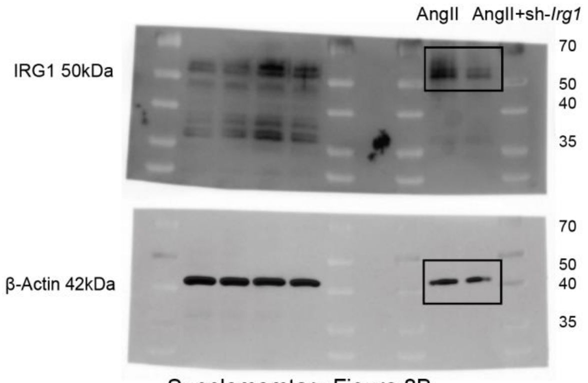

Supplememtary Figure 8A

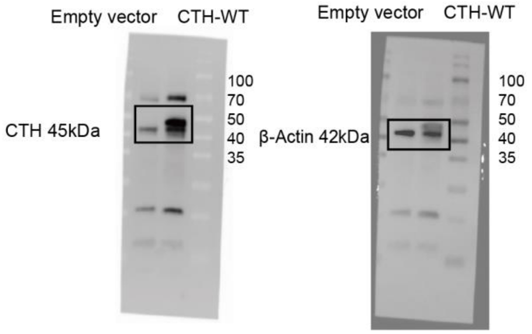

Supplememtary Figure 8B

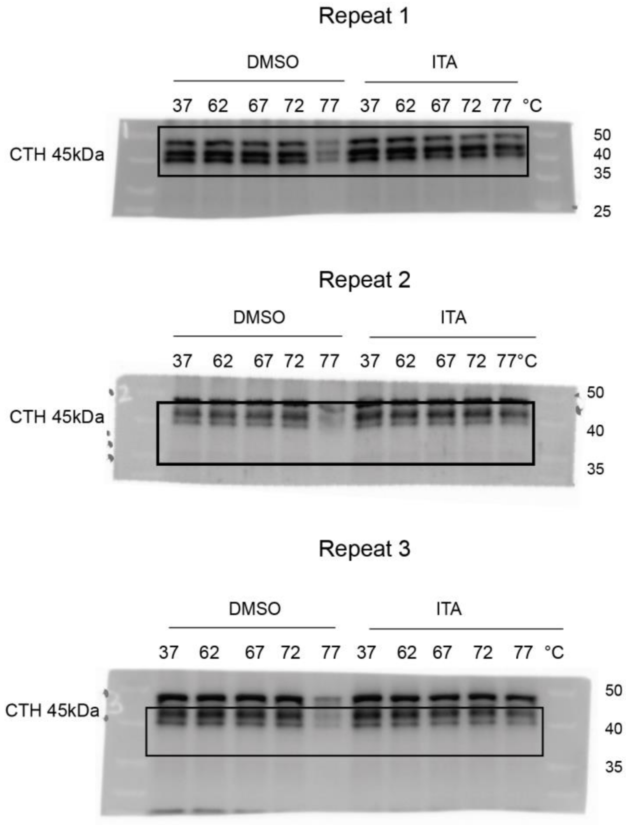

Supplememtary Figure 8C

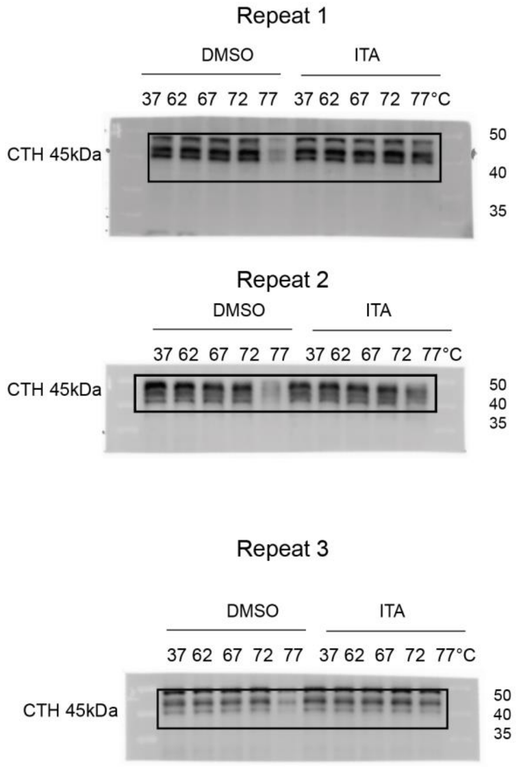

Supplementary figure 10

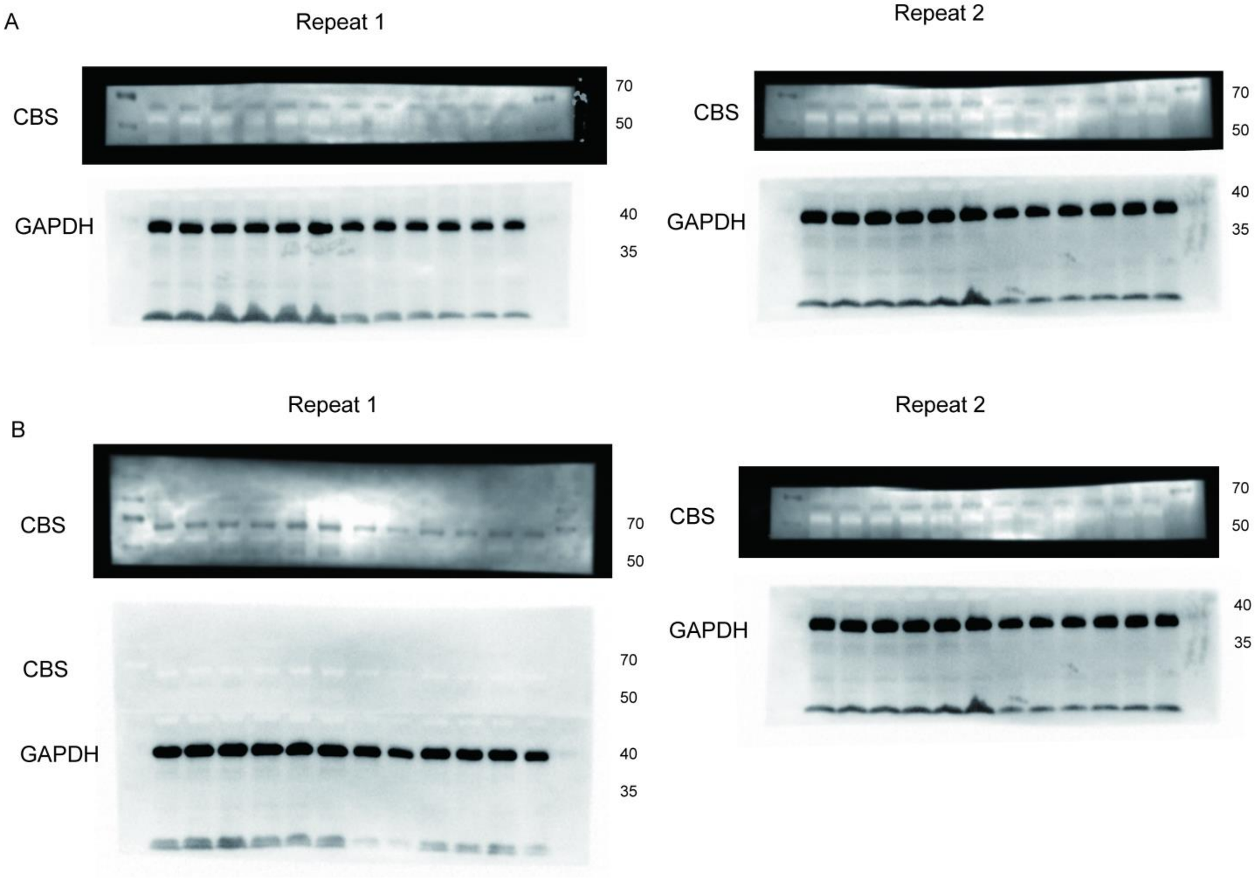

Supplement: Supplementary file 1 [file jcdd-13-00338-s001.zip › Raw blots.pdf]
