# Supplementary material for: Immunoresponsive Gene 1–Itaconate Exacerbates Hypertension by Inhibiting the Cystathionine Gamma-Lyase/Hydrogen Sulfide Pathway
Source: J Cardiovasc Dev Dis. 2026 Jul 20;13(7):338. doi: 10.3390/jcdd13070338 (PMC13410097; doi:10.3390/jcdd13070338)
Supplement: Supplementary file 1 [file jcdd-13-00338-s001.zip › S1 Supplementary_Material V10 -clean.pdf]

Supplementary Material

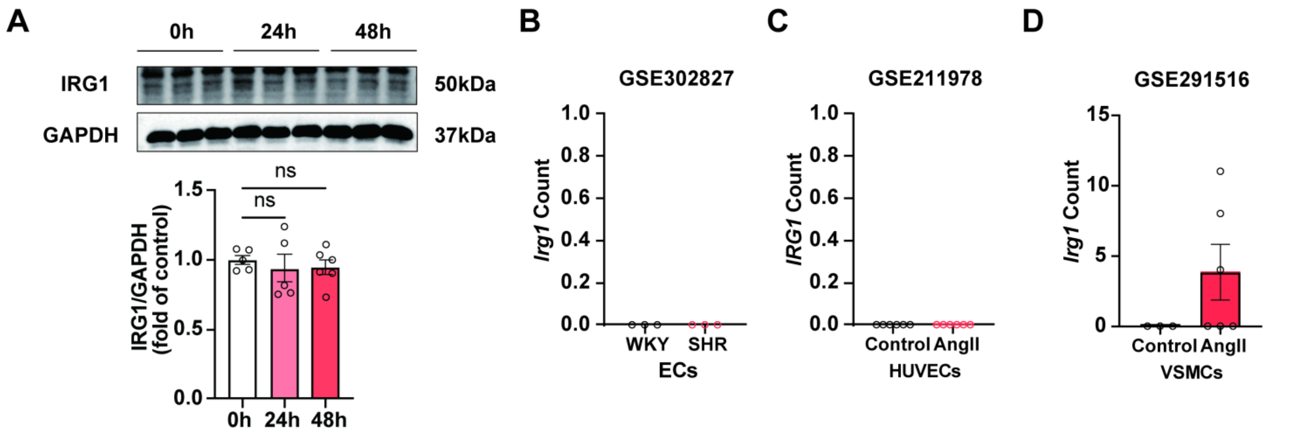

Figure S1. IRG1 is not induced in endothelial cells but is upregulated in VSMCs under hypertensive conditions or Ang II stimulation. (A) Representative Western blots and quantitative analysis of IRG1 protein in HUVECs treated with Ang II for 0, 24, and 48 h (*n* = 5–6). (B-D) Analysis of *Irg1*/*IRG1* mRNA expression in endothelial cells (B, C) and VSMCs (D) under hypertensive conditions or Ang II stimulation, using public GEO datasets GSE302827 (B, *n* = 3 per group), GSE211978 (C, *n* = 6 per group), and GSE291516 (D, *n* = 3 or 6 per group). Data are presented as the mean ± SEM. Statistical comparisons were performed using two-tailed Student t-test for comparisons between two groups, and one-way ANOVA for comparisons among three groups. ns, $P > 0.05$. Abbreviations: IRG1/*Irg1*, immunoresponsive gene 1; GAPDH, glyceraldehyde-3-phosphate dehydrogenase; EC, endothelial cell; VSMC, vascular smooth muscle cell; HUVEC, human umbilical vein endothelial cell; WKY, Wistar-Kyoto; SHR, spontaneously hypertensive rat.

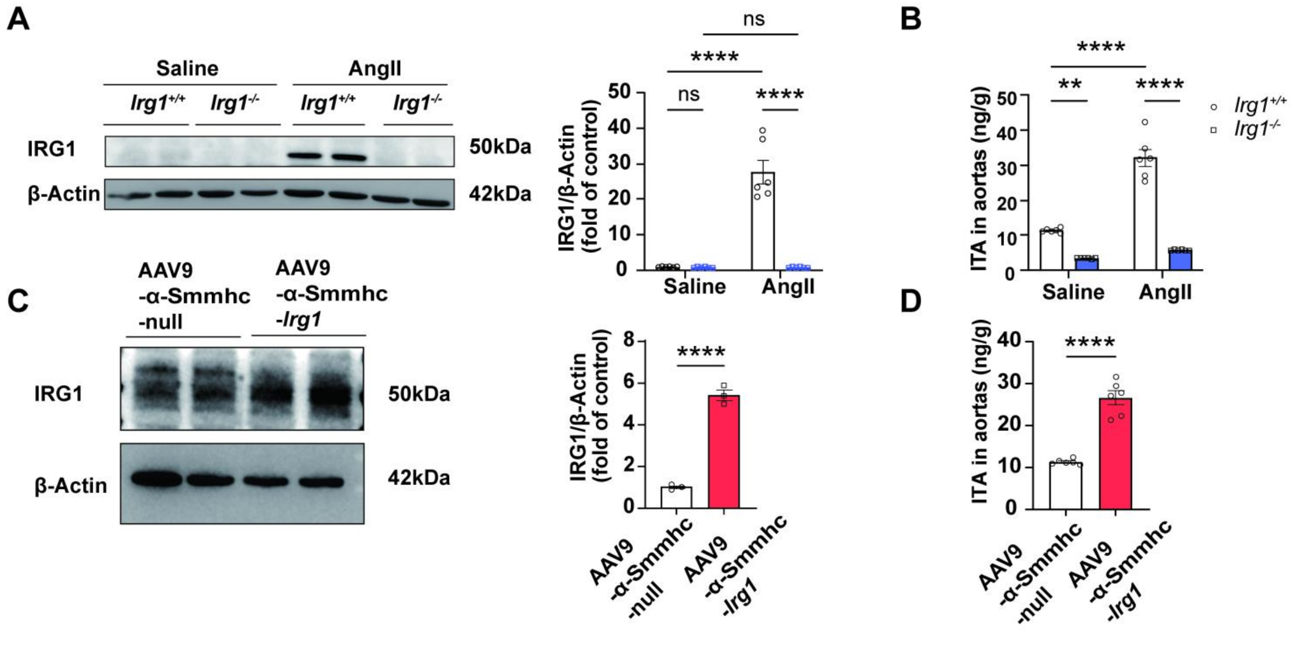

Figure S2. Validation of *Irg1* knockout and overexpression efficiency in mouse aortas. (A) Representative Western blot images and statistical analysis of IRG1 expression in aortas from $Irg1^{+/+}$ and $Irg1^{-/-}$ mice with or without Ang II infusion for 14 days (n = 6 per group). (B) ITA levels in mouse aortas were measured using an ITA ELISA kit (n = 6 per group). (C) Representative Western blot images and statistical analysis of IRG1 expression in aortas from AAV9-α-Smmhc-null and AAV9-α-Smmhc-*Irg1* mice with or without Ang II infusion for 14 days (n = 3 per group). (D) ITA levels in mouse aortas were measured using an ITA ELISA kit (n = 6 per group). Data are presented as the mean ± SEM. Data were analyzed using two-tailed Student t-test for comparisons between two groups, and two-way ANOVA followed by Tukey's multiple comparisons test for comparisons among three or more groups. ns, $P > 0.05$; $^{**}P < 0.01$; $^{****}P < 0.0001$. Abbreviations: IRG1/Irg1: immunoresponsive gene 1; ITA: itaconate; AAV9: adeno-associated virus serotype 9; α-Smmhc: alpha-smooth muscle myosin heavy chain; ELISA, enzyme-linked immunosorbent assay; SEM, standard error of the mean.

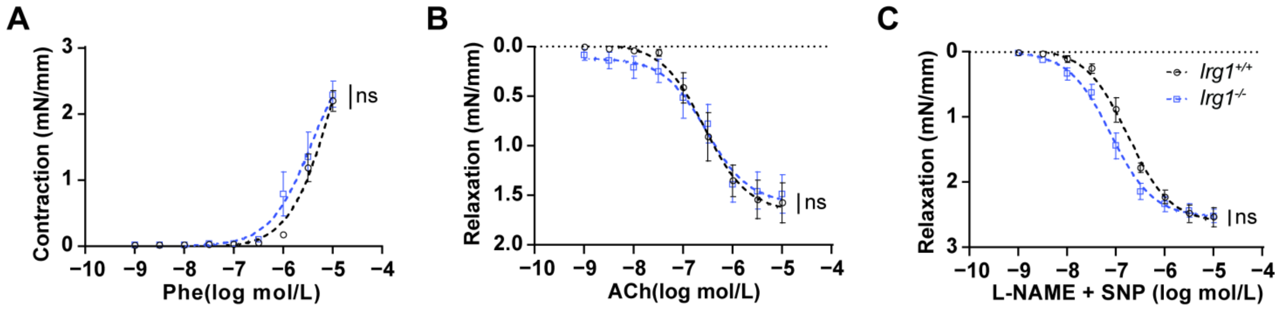

Figure S3. *Irg1* knockout does not affect resistance artery function in normotensive mice. (A–C) Concentration-response curves for contraction (A), endothelium-dependent relaxation (B), and endothelium-independent relaxation (C) in mesenteric resistance arteries from *Irg1*$^{+/+}$ and *Irg1*$^{-/-}$ mice under basal conditions ($n = 6$ per group). Data are presented as the mean ± SEM. For the vascular ring concentration-response curves in panels A–C, the trapezoidal rule was used to compute the AUC of each arterial segment, and the resulting AUC values were compared between the two groups using unpaired Student's *t*-test. ns, $P > 0.05$. Abbreviations: Phe, phenylephrine; ACh, acetylcholine; L-NAME, Nω-nitro-L-arginine methyl ester; SNP, sodium nitroprusside; *Irg1*$^{+/+}$, *Irg1* WT; *Irg1*$^{-/-}$, *Irg1* knockout; AUC, area under the curve; SEM, standard error of the mean.

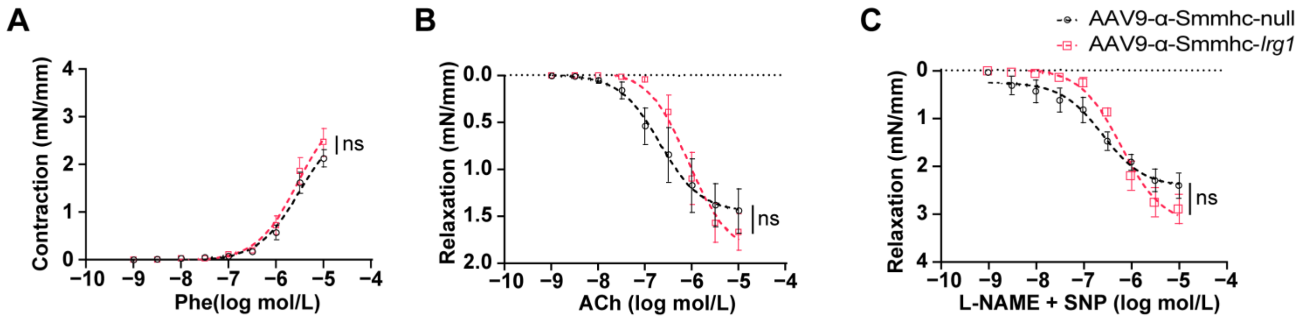

Figure S4. SMC-specific *Irg1* overexpression does not affect resistance artery function in normotensive mice. (A–C) Concentration-response curves for contraction (A), endothelium-dependent relaxation (B), and endothelium-independent relaxation (C) in mesenteric resistance arteries from AAV9-α-Smmhc-null and AAV9-α-Smmhc-*Irg1* mice under basal conditions ($n = 6$ per group). Data are presented as the mean ± SEM. For the vascular ring concentration-response curves in panels A–C, the trapezoidal rule was used to compute the AUC of each arterial segment, and the resulting AUC values were compared between the two groups using unpaired Student's t-test. ns, $P > 0.05$. Abbreviations: Phe: phenylephrine; ACh: acetylcholine; L-NAME: Nω-nitro-L-arginine methyl ester; SNP: sodium nitroprusside; AAV9: adeno-associated virus serotype 9; α-Smmhc: alpha-smooth muscle myosin heavy chain; *Irg1*: immunoresponsive gene 1; AUC, area under the curve; SEM, standard error of the mean.

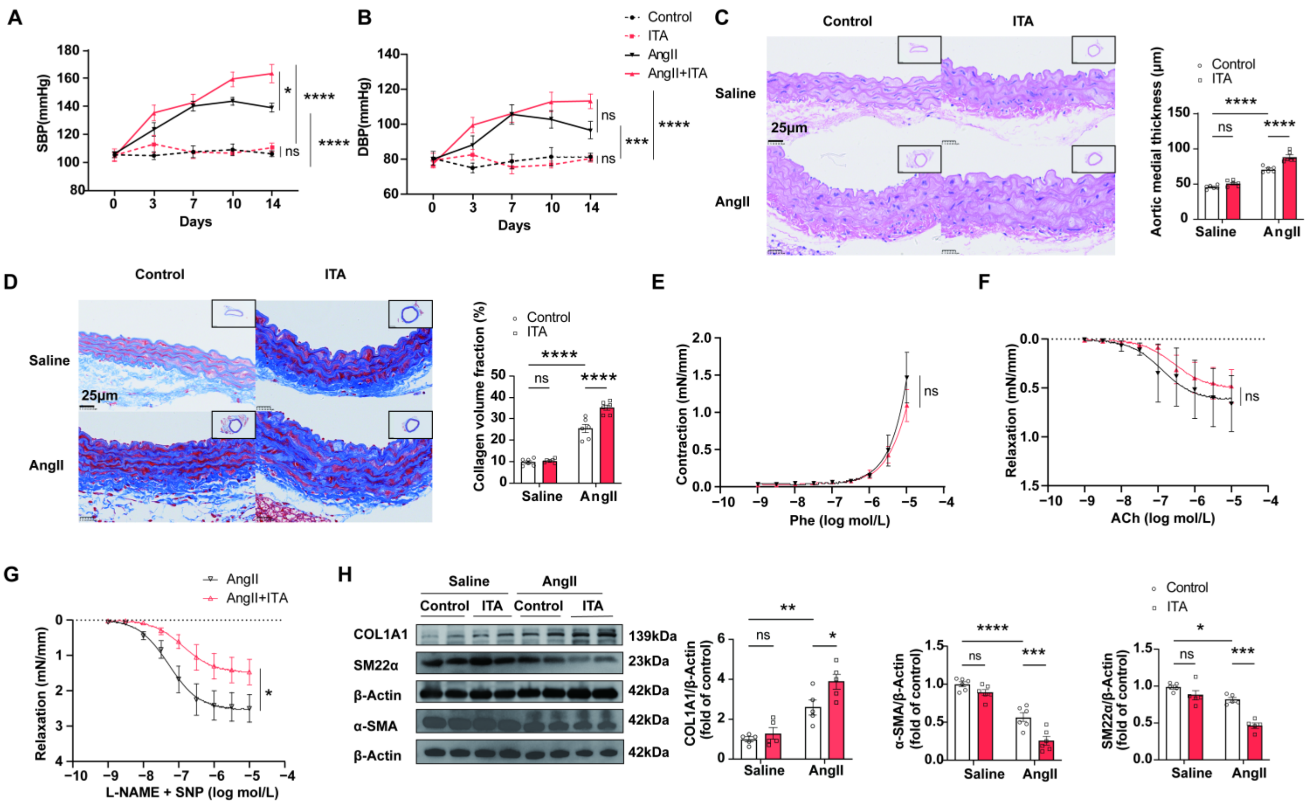

Figure S5. Intraperitoneal injection of ITA exacerbates elevated blood pressure, vascular remodeling, and vascular dysfunction in hypertensive mice. (A–B) SBP (A) and DBP (B) in saline-treated and ITA-treated mice infused with or without Ang II for 14 days ($n = 6$–10). (C–D) Representative H&E (C) and Masson's trichrome staining (D) images, and statistical analysis of aortic medial thickness and collagen deposition in thoracic aortas from control mice and ITA-treated mice treated with saline or Ang II for 14 days ($n = 6$). (E–G) Concentration-response curves of contraction (E), endothelium-dependent relaxation (F), and endothelium-independent relaxation (G) in mesenteric arteries from vehicle control mice and ITA-treated mice infused with Ang II for 14 days ($n = 6$). (H) Expression levels of COL1A1, α-SMA, and SM22α in aortas from mice treated with or without ITA and/or Ang II for 14 days ($n = 5$–6). Data are presented as mean ± SEM. Comparisons between two groups were analyzed by two-tailed Student's t-test. Multiple comparisons among groups were performed by two-way ANOVA followed by Tukey's post hoc test. AUC values for concentration-response curves (E–G) were compared by unpaired Student's t-test. ns, $P > 0.05$; *$P < 0.05$; **$P < 0.01$; ***$P < 0.001$; ****$P < 0.0001$. Abbreviations: SBP: systolic blood pressure; DBP: diastolic blood pressure; Ang II: angiotensin II; Phe: phenylephrine; ACh: acetylcholine; L-NAME: Nω-nitro-L-arginine methyl ester; SNP: sodium nitroprusside; COL1A1: collagen 1α1; α-SMA: α-smooth muscle actin; SM22α: smooth muscle 22 alpha; ITA: itaconate; AUC, area under the curve; SEM, standard error of the mean.

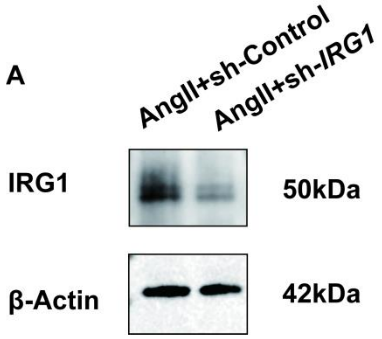

Figure S6. Validation of sh-*IRG1* knockdown efficiency in HASMCs. (A) HASMCs were transfected with sh-Control or sh-*IRG1*, then stimulated with Ang II (1×10^{-6} M) for 48 h. Western blotting showed that IRG1 protein level was markedly decreased in the sh-*IRG1* group under Ang II treatment, indicating successful knockdown of IRG1. β-Actin was used as a loading control. Abbreviations: IRG1, immunoresponsive gene 1; HASMC, human aortic smooth muscle cell; Ang II, angiotensin II; sh-*IRG1*, short hairpin RNA targeting IRG1; SEM, standard error of the mean.

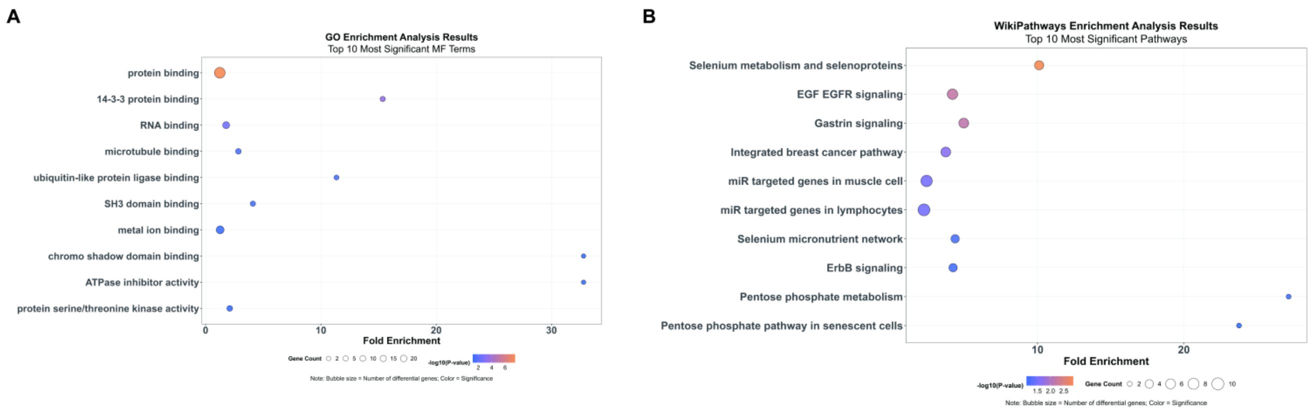

Figure S7. Pathway enrichment analysis of TPP. (A–B) Top 10 enriched GO (A) and WikiPathways (B) terms for the top 200 differential proteins with significantly increased thermal stability following ITA treatment (ratio ≥ 1.2, $P < 0.05$), as identified by TPP analysis in HASMCs. Abbreviations: TPP, thermal proteomic profiling; ITA, itaconate; GO, Gene Ontology; HASMC, human aortic smooth muscle cell.

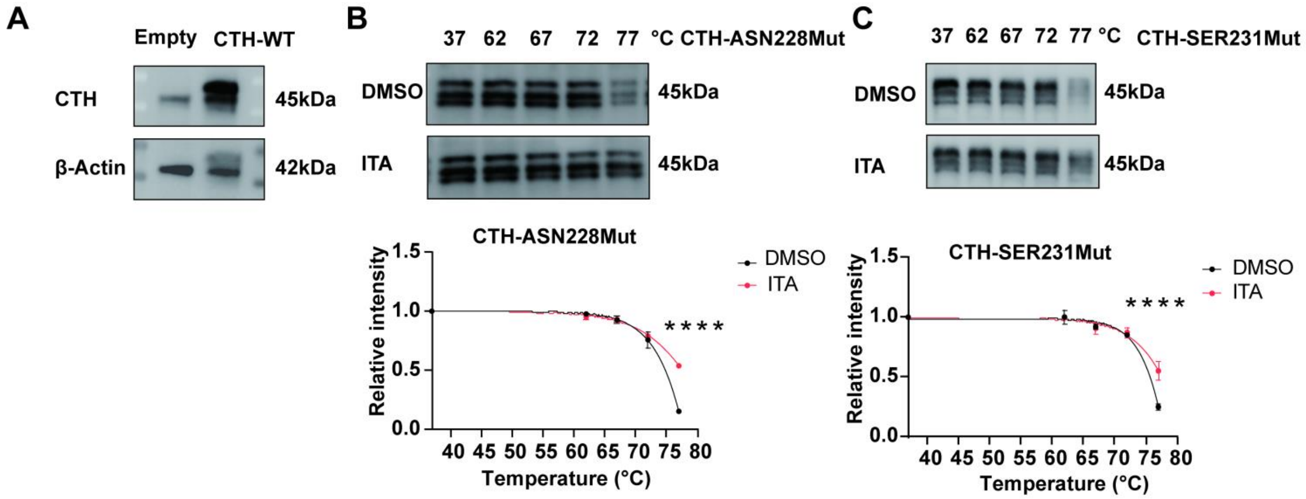

Figure S8. Following validation of CTH overexpression plasmid transfection in HEK293A cells, CETSA was performed to analyze ITA binding to site-mutated CTH. (A) Validation of successful transfection of CTH overexpression plasmid in HEK293A cells. (B) CETSA assay was performed to detect the binding between ITA and CTH after transfection of CTH-ASN228 site mutant plasmid into HEK293A cells ($n = 3$). (C) CETSA assay was performed to detect the binding between ITA and CTH after transfection of CTH-SER231 site mutant plasmid into HEK293A cells ($n = 3$). Comparisons between two groups were analyzed by unpaired Student's t-test. Data are presented as mean ± SEM. ****$P < 0.0001$. Abbreviations: ITA, itaconate; CTH-SER231Mut, Cystathionine gamma-lyase Serine 231 mutant; CTH-ASN228Mut, Cystathionine gamma-lyase Asparagine 228 mutant.

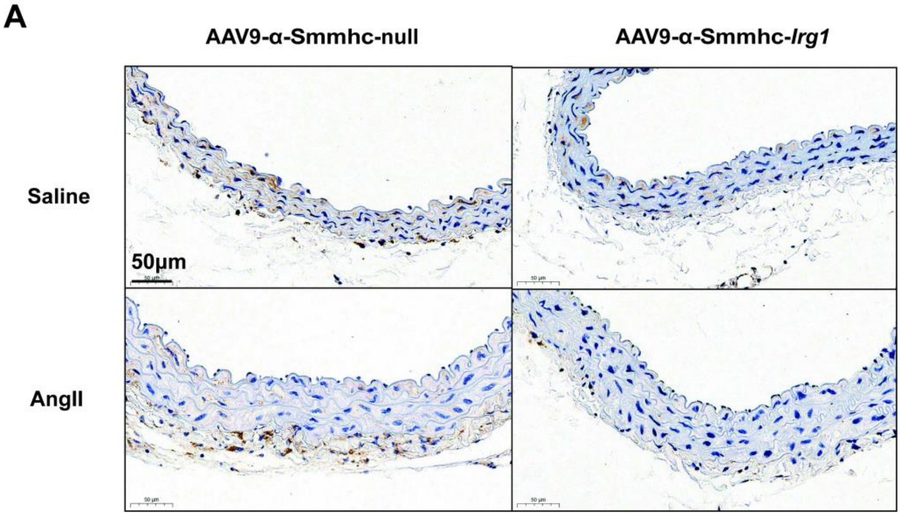

Figure S9. SMC-specific *Irg1* overexpression aggravates Ang II-induced downregulation of aortic CTH protein. (A) Representative immunohistochemistry micrographs and quantitative mean optical density analysis of CTH in mouse aortas transduced with AAV9-α-Smmhc-null or AAV9-α-Smmhc-*Irg1*, followed by saline or Ang II infusion. Brown signals indicate CTH immunoreactivity; nuclei are stained blue. Scale bar, 50 μm. Quantification data are normalized to saline-treated AAV9-α-SMMHC-null controls. ns, $P > 0.05$; **$P < 0.01$; ***$P < 0.001$, $n = 5$ per group. Abbreviations: AAV9, adeno-associated virus serotype 9; α-SMMHC, α-smooth muscle myosin heavy chain; Ang II, angiotensin II; CTH, cystathionine gamma-lyase; *Irg1*, immunoresponsive gene 1; SMC, smooth muscle cell.

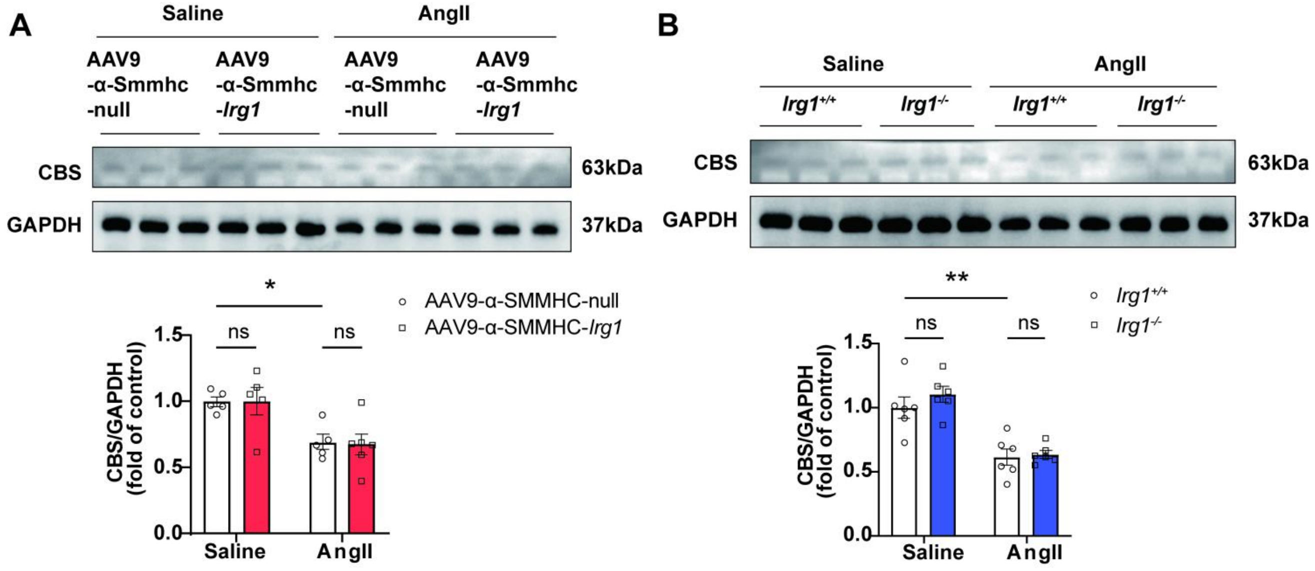

Figure S10. CBS expression in mouse aorta is not regulated by IRG1. (A) Western blot and quantitative densitometry of CBS in aorta from mice with SMC-specific *Irg1* overexpression (AAV9-α-Smmhc-*Irg1*) or empty vector control (AAV9-α-Smmhc-null) under saline or Ang II treatment (n = 5–6). GAPDH was used as loading control. (B) Western blot and quantitative densitometry of CBS in aorta from $Irg1^{+/+}$ and $Irg1^{-/-}$ mice with saline or Ang II infusion (n = 6). Data are presented as mean ± SEM. Multiple comparisons among groups were performed by two-way ANOVA followed by Tukey's post-hoc test. ns, $P > 0.05$; $*P < 0.05$; $**P < 0.01$. Abbreviations: CBS, cystathionine β-synthase; GAPDH, glyceraldehyde-3-phosphate dehydrogenase; Ang II, angiotensin II; AAV9, adeno-associated virus serotype 9; α-SMMHC, α-smooth muscle myosin heavy chain; $Irg1^{+/+}$, *Irg1* WT; $Irg1^{-/-}$, *Irg1* knockout.

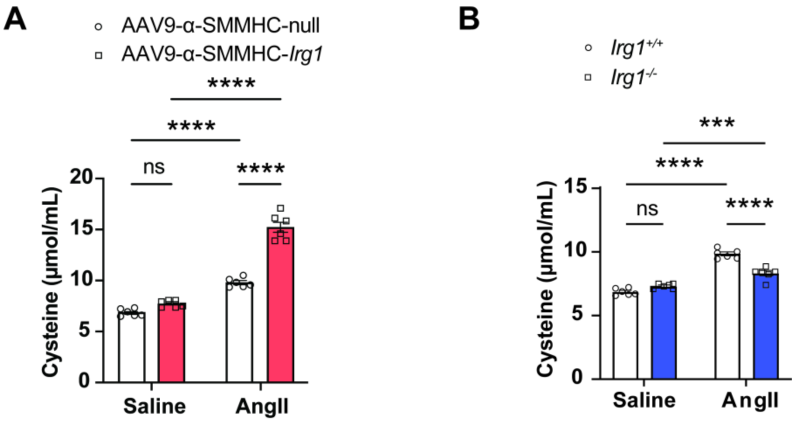

Figure S11. Circulating Cysteine levels are regulated by IRG1 during Ang II-induced hypertension. (A) Plasma L-cysteine concentrations in mice with SMC-specific *Irg1* overexpression (AAV9-α-Smmhc-*Irg1*) or empty vector control under saline or Ang II infusion ($n = 6$). (B) Plasma cysteine quantification in *Irg1*$^{+/+}$ and *Irg1*$^{-/-}$ mice with saline or Ang II treatment ($n = 6$). Basal cysteine levels were unchanged between genotypes (ns). Data are shown as mean ± SEM. Statistical analysis was conducted via two-way ANOVA followed by Tukey's post-hoc test for all pairwise comparisons. ns, $P > 0.05$; ***$P < 0.001$; ****$P < 0.0001$.

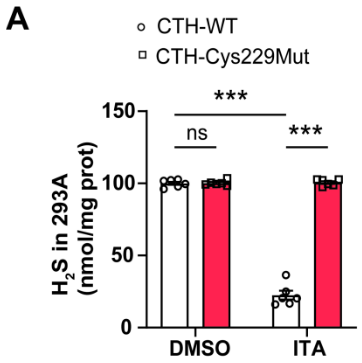

Figure S12. ITA inhibits CTH-mediated H_2S production by targeting the Cys229 residue. (A) H_2S production in HEK293A cells expressing CTH-WT or CTH-229Mut treated with DMSO or ITA. ITA significantly inhibited H_2S production only in cells expressing CTH-WT, whereas the C229 mutation abrogated this effect (n = 6). Data are presented as mean ± SEM. Multiple comparisons among groups were performed by two-way ANOVA followed by Tukey's multiple comparisons test. ns, $P > 0.05$; ***$P < 0.001$. Abbreviations: H_2S, hydrogen sulfide; ITA, itaconate; CTH-WT: wild-type cystathionine gamma-lyase; CTH-Cys229Mut: CTH cysteine 229 mutant.
